# Cyanobacteria and Red Macroalgae as Potential Sources of Antioxidants and UV Radiation-Absorbing Compounds for Cosmeceutical Applications

**DOI:** 10.3390/md18120659

**Published:** 2020-12-21

**Authors:** Julia Vega, José Bonomi-Barufi, Juan Luis Gómez-Pinchetti, Félix L. Figueroa

**Affiliations:** 1 Institute of Blue Biotechnology and Development (IBYDA), Ecology Department, Campus Universitario de Teatinos s/n, University of Malaga, 29071 Malaga, Spain; juliavega@uma.es; 2 Botany Department, Campus of Trindade, Florianópolis, Federal University of Santa Catarina, Santa Catarina 88040-970, Brazil; jose.bonomi@ufsc.br; 3 Banco Español de Algas (BEA), Institute of Oceanography and Global Change (IOCAG), University of Las Palmas de G.C., Muelle de Taliarte s/n, 35214 Telde, Spain; juan.gomez@ulpgc.es

**Keywords:** antioxidant activity, cyanobacteria, mycosporine-like amino acids, scytonemin, photoprotection, red macroalgae, UV-screen

## Abstract

In recent years, research on natural products has gained considerable attention, particularly in the cosmetic industry, which is looking for new bio-active and biodegradable molecules. In this study, cosmetic properties of cyanobacteria and red macroalgae were analyzed. The extractions were conducted in different solvents (water, ethanol and two combinations of water:ethanol). The main molecules with antioxidant and photoprotective capacity were mycosporine-like amino acids (MAAs), scytonemin and phenolic compounds. The highest contents of scytonemin (only present in cyanobacteria) were observed in *Scytonema* sp. (BEA 1603B) and *Lyngbya* sp. (BEA 1328B). The highest concentrations of MAAs were found in the red macroalgae *Porphyra umbilicalis*, *Gelidium corneum* and *Osmundea pinnatifida* and in the cyanobacterium *Lyngbya* sp. *Scytonema* sp. was the unique species that presented an MAA with maximum absorption in the UV-B band, being identified as mycosporine-glutaminol for the first time in this species. The highest content of polyphenols was observed in *Scytonema* sp. and *P. umbilicalis*. Water was the best extraction solvent for MAAs and phenols, whereas scytonemin was better extracted in a less polar solvent such as ethanol:_d_H_2_O (4:1). Cyanobacterium extracts presented higher antioxidant activity than those of red macroalgae. Positive correlations of antioxidant activity with different molecules, especially polyphenols, biliproteins and MAAs, were observed. Hydroethanolic extracts of some species incorporated in creams showed an increase in the photoprotection capacity in comparison with the base cream. Extracts of these organisms could be used as natural photoprotectors improving the diversity of sunscreens. The combination of different extracts enriched in scytonemin and MAAs could be useful to design broad-band natural UV-screen cosmeceutical products.

## 1. Introduction

Solar UV radiation (UVR) comprises UV-C (200–280 nm), UV-B (280–315 nm) and UV-A (315–400 nm), although only UV-A and a small part of UV-B reach the Earth’s surface. UV-B radiation is the most harmful, inducing mutations in the DNA of skin cells, whereas UV-A radiation is indirectly mutagenic by generating reactive oxygen species (ROS) [1,2]. UVR can provoke some clinical effects in humans, such as erythema, pigmentation, immunosuppression, photoaging or carcinogenesis [3,4,5,6]. Recently, several studies have demonstrated also the biological effects of blue light (400–450 nm) in human skin, such as pigmentation, erythema or free radical production [7,8,9].

UV filters included in sunscreens are divided in synthetic organic (e.g., oxybenzone, octinoxate), based on the capacity to absorb UVR, and inorganic or physical (e.g., titanium dioxide (TiO_2_) and zinc oxide (ZnO)), which reflect UV rays. Synthetic organic filters are suspected to provoke some negative effects in humans, such as allergic reactions, photo-toxicity or endocrine disruptions [10]. UV filters can also be accumulated in the aquatic environment causing negative impacts, such as bleaching of coral reefs or hormone disorders in mammals [11,12]. The development of biological photoprotectors of broad band spectrum that filter UV-B, UV-A, blue light and infrared, as well as provide antioxidant activity, is a very active research area [13]. Natural photoprotectors are more beneficial for health and the environment due to their low toxicity and biodegradable character [14].

Research on natural products has gained considerable attention, especially in the cosmetic industry, which nowadays is growing in a global scale [15]. Aquatic organisms, specially algae and cyanobacteria, have acquired great biotechnological interest in this area due to their high content of molecules presenting antioxidant, antimicrobial, anti-inflammatory, immuno-stimulant and photoprotective properties [16,17,18] that can be beneficial for the skin health. Among natural compounds presented in aquatic organism, mycosporine-like amino acids (MAAs), scytonemin, carotenoids, phycobiliproteins and polyphenols are becoming promising due to their UVR-blue light screen properties and/or potential antioxidant activity.

Cyanobacteria, also known as blue-green algae, belong to a group of photosynthetic prokaryotes, distributed in many habitats and adapted to extreme conditions such as deserts, illuminated caves, salt lakes or polar regions [19]. Macroalgae are more complex organisms distributed in coastal areas around the world. Species from the intertidal normally live in a harsh environment with high radiation, temperature fluctuations, changes in the salinity, hydrodynamics or desiccation periods [20].

An immediate response of algae against stress conditions is an excessive production of ROS [21], which can produce DNA mutations, proteins denaturalization or lipid peroxidation [22], affecting important biological processes such as photosynthesis. Different authors demonstrated that UVR could negatively affect algal photosynthesis, growth or spores survival [23,24,25]. Thus, algae have developed protection mechanisms against the oxidative stress and the exposure to high UVR. An antioxidant defense system can be enzymatic (e.g., superoxide dismutase or catalase) or non-enzymatic (e.g., vitamin E, carotenoids or phycobiliproteins) [26], and two of the most important molecules with the capacity to dissipate the UVR as heat in cyanobacteria and red algae are MAAs and scytonemin (only present in cyanobacteria).

The sun protection factor (SPF) measures the protection of a sunscreen against UV rays, but this factor is only based on the efficacy to prevent erythema, which is mainly related to UV-B radiation [27]. The SPF can be measured in vitro using the erythematic action spectrum [28,29]. More recently, the in vitro measurement of UVA protection factor (UVAPF) was proposed using the persistent pigment darkening (PPD) action spectrum [30]. However, there are other effects of the UVR on the skin, such as elastosis, photoaging or immunosuppression [31,32,33]. De la Coba et al. suggested the biological effective protection factors (BEPFs), which can be related to different action spectra [34]. Another index suggested by Schneider et al. is the effective solar absorpted radiation (ESAR), which can also be related to different action spectra and measures the capacity of sunscreens to absorb the effective UV radiation [35].

The aim of this study was to evaluate the antioxidant and photoprotective properties of several cyanobacteria and red macroalgae, using compatible solvents with natural cosmetics for the extraction processes. Contents of MAAs, polyphenols, phycobiliproteins and scytonemin, among others, were evaluated in all species assayed.

## 2. Results

### 2.1. UV Absorption Spectra

**Figure 1:**
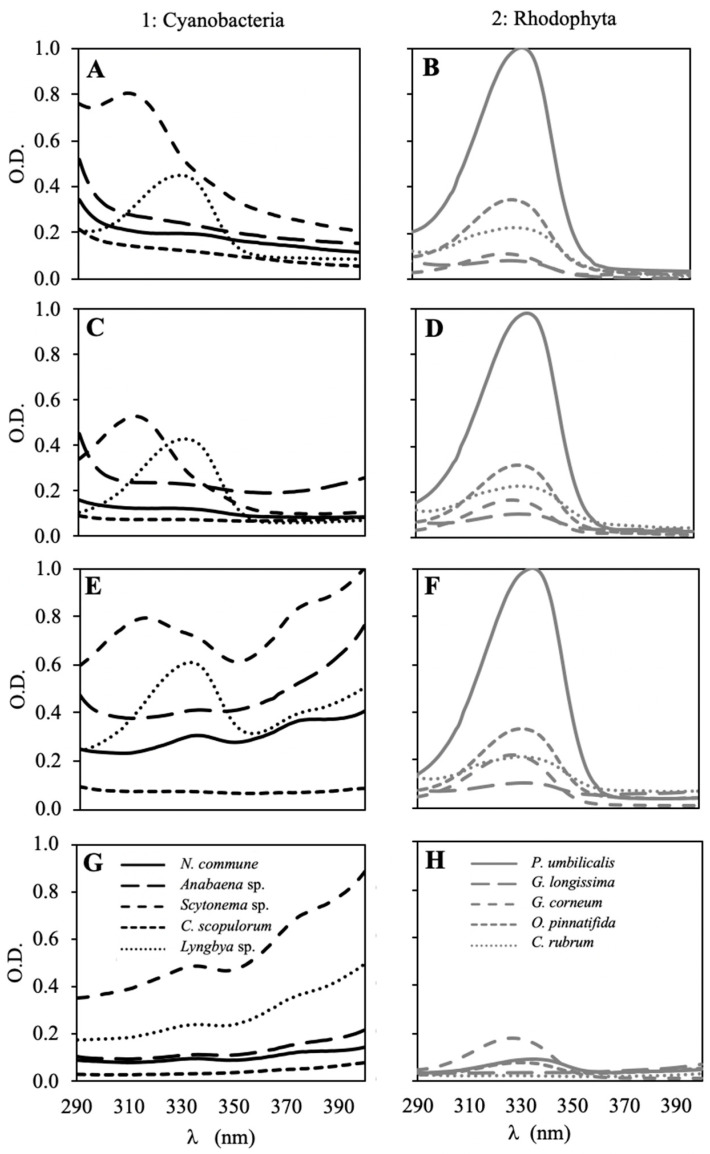
Optical density in the UV region of the spectra (λ = 290–400 nm) of the different algal species (1: cyanobacteria, dark lines; 2: Rhodophyta, grey lines) after extraction in different solvents: _d_H_2_O (**A**,**B**), ethanol:_d_H_2_O (1:1) (**C**,**D**), ethanol:_d_H_2_O (4:1) (**E**,**F**), and ethanol (**G**,**H**). Data are an average of three measurements and the curves were normalized from 0 to 1 for cyanobacteria and red macroalgae separately. The same algal concentration (30 mg DW mL^−1^) was used to obtain all measurements of the extracts.

The absorption spectra in the UV region of all the species extracted in the different solvents are shown in Figure 1. Most of the species showed peaks in this region. In the case of cyanobacteria species, only two strains presented prominent peaks, *Scytonema* sp. showed a peak with a maximum absorbance (λ_max_) around 310–314 nm and *Lyngbya* sp. presented a peak with a λ_max_ around 330–333 nm. These peaks were similar in all of the solvents assayed (Figure 1A,C,E), except in pure ethanol (Figure 1G). Other peaks with λ_max_ at 335–337 nm and 375–378 nm were only visible in two solvents (ethanol:_d_H_2_O (4:1) and ethanol), and mainly in *Scytonema* sp. and *Lyngbya* sp., but they could also be appreciated in *Nostoc commune* and *Anabaena* sp. (Figure 1E,G). In red algae, all species showed peaks with λ_max_ around 330–335 nm; the highest peak was observed in *P. umbilicalis* and the lowest in *G. longissima*. These peaks were also similar in three of the solvents (Figure 1B,D,F), and only in ethanol the peak was much lower (Figure 1H).

### 2.2. Biochemical Composition

The contents of total internal carbon and nitrogen, proteins, carbohydrates, lipids and the C:N ratios of the different species are shown in Table 1. In most of the cases, significant differences were observed among species (*p* < 0.05) (Table S1A). In cyanobacteria, the highest internal carbon content (>400 mg g^−1^ DW) was observed in *Scytonema* sp., *N. commune* and *Anabaena* sp., and the lowest content was obtained in *C. scopulorum* (313 mg g^−1^ DW). The nitrogen concentration was also higher in *Anabaena* sp. and *Scytonema* sp. (85 and 62 mg g^−1^ DW, respectively) and lower in *C. scopulorum* (35 mg g^−1^ DW). The highest C:N ratio was reached in *C. scopulorum* (9.1) and the lowest in *Anabaena* sp. (8.5). The highest total protein content estimated from the N content was observed in *Anabaena* sp. and the lowest in *C. scopulorum* (407 and 165 mg g^−1^ DW, respectively), whereas the highest soluble protein content was reached in *N. commune* (71 mg g^−1^ DW) and the lowest in *C. scopulorum* (8 mg g^−1^ DW). The highest levels of carbohydrates were observed in *N. commune* (40.8%) and *Lyngbya* sp. (408 and 334 mg g^−1^ DW, respectively), and the lowest amount was obtained in *Anabaena* sp. and *Scytonema* sp. (185 and 196 mg g^−1^ DW, respectively), in spite of the presence of a high internal carbon content. *N. commune* presented the highest lipid content and *Anabaena* sp. the lowest (21 and 16 mg g^−1^ DW, respectively).

In red macroalgae, the highest C content was observed in *G. corneum* and *P. umbilicalis* (351 and 340 mg g^−1^ DW, respectively) and the lowest in *G. longissima* (256 mg g^−1^ DW). The N content was lower than the observed in cyanobacteria, being the highest concentration obtained in *P. umbilicalis* and the lowest in *O. pinnatifida* (45 and 25 mg g^−1^ DW). The C:N ratio was higher in *G. corneum* and *O. pinnatifida* (10.6 and 9.2, respectively) and the lowest in *C. rubrum* (6.7). Total protein contents were also the highest in *P. umbilicalis* and the lowest in *O. pinnatifida* (220 and 123 mg g^−1^ DW). Soluble proteins followed the same pattern; the highest content was observed in *P. umbilicalis* and the lowest in *O. pinnatifida* (34 and 6 mg g^−1^ DW). The highest carbohydrate content was observed in *P. umbilicalis* and *G. corneum* (318 and 275 mg g^−1^ DW, respectively) and the lowest in *C. rubrum* (139 mg g^−1^ DW). Macroalgae presented low lipid contents; all species showed less than 1%.

The results of phycobiliprotein and scytonemin contents in the different species are shown in Table 2. In most of the cases, significant differences were observed among species (*p* < 0.05) (Table S1). Both groups of tested organisms presented biliproteins, although different composition and concentrations were observed. Cyanobacteria normally presented more phycocyanin (PC) than phycoerythrin (PE), whereas red macroalgae were enriched mostly in PE. *N. commune* presented the highest content of PE (14 mg g^−1^ DW), followed by *Scytonema* sp. (3.7 mg g^−1^ DW). The highest content of phycocyanin was also observed in *N. commune* (25mg g^−1^ DW) and *Scytonema* sp. (19mg g^−1^ DW). *C. scopulorum* showed a very low content of biliproteins. In red algae, *P. umbilicalis* presented the highest content of PE (3.4 mg g^−1^ DW), and *O. pinnatifida* the lowest (0.07 mg g^−1^ DW). In general, phycocyanin contents were low in all red algal species.

Considering the cyanobacteria, the higher contents of scytonemin were found in *Scytonema* sp. and *Lyngbya* sp. (2.2 and 1.2 mg g^−1^ DW, respectively). In the other three species, the contents were significantly much lower (<0.5 mg g^−1^ DW).

**Figure 2:**
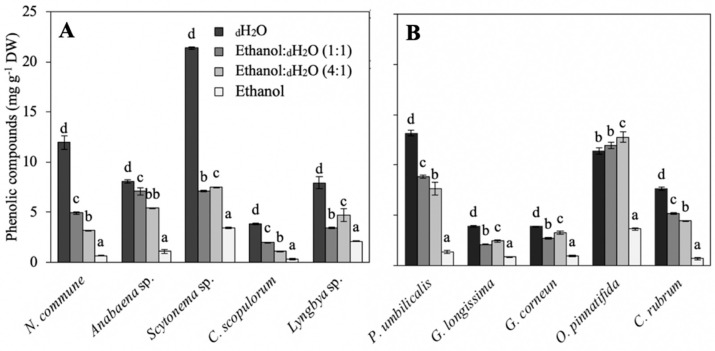
Phenolic compounds concentration (mg g^−1^ DW) of the different cyanobacteria (**A**) and red algae (**B**) extracted in different solvents (_d_H_2_O, ethanol:_d_H_2_O (1:1), ethanol:_d_H_2_O (4:1) and ethanol), and expressed as average ± standard deviation (SD) (*n* = 3). Different letters indicate significant differences among solvents for each specie (ANOVA, *p* < 0.05, SNK test).

The phenolic compound contents of the different species are shown in Figure 2. Significant differences were observed among species and among solvents (*p* < 0.05) (Table S1). In most of the cases (83%), the best solvent for the extraction of phenolic compounds was _d_H_2_O. In cyanobacteria (Figure 2A), the higher contents were observed in the aqueous extracts of *Scytonema* sp. and *N. commune* (22.5 and 13 mg g^−1^ DW, respectively). In red algae, the aqueous extract of *P. umbilicalis* and the ethanol:_d_H_2_O (4:1) of *O. pinnatifida* (aprox. 13 mg g^−1^ DW in both) showed the highest values (Figure 2B). The lowest content was observed in *C. scopulorum*, *G. longissima* and *G. corneum*, and in all cases, ethanol was not an effective solvent for the extraction of these compounds.

The quantitative and qualitative MAA contents are shown in Table 3. In relation to the total MAAs content, significant differences were observed among species (*p* < 0.05) (Table S1). Only two species of cyanobacteria showed relevant concentration of these molecules (*Scytonema* sp. and *Lyngbya* sp.), whereas all the red algae presented significant amounts of MAAs. In cyanobacteria, *Lyngbya* sp. showed the highest content (1.8 mg g^−1^ DW), whereas in red algae, the highest content of MAAs (5.2 mg g^−1^ DW) was observed in *P. umbilicalis*, followed by *G. corneum* and *O. pinnatifida* (1.8 and 1.5 mg g^−1^ DW, respectively). Considering the different types of MAAs detected using HPLC and ESI-MS, *Scytonema* sp. presented a unique peak at 310 nm (identified as mycosporine-glutaminol). *Lyngbya* sp. presented a predominant peak at 330 nm (asterina-330, >85%) and a small peak at 320 nm (palythine). *P. umbilicalis* showed the highest peak at 334 nm (porphyra-334, >85%) and other peaks at 320, 330, 332 and 333 nm (palythine, asterina-330, palythinol and shinorine, respectively). *G. longissima* only presented a peak at 332 nm (shinorine). *G. corneum* presented a predominant peak at 330 nm (asterina-330, >60%) and two other peaks at 320 and 332 nm (palythine and shinorine, respectively). *C. rubrum* presented different peaks at 320, 332 and 340 nm (palythine, shinorine and an unknown absorbing compound, respectively).

### 2.3. Antioxidant Activity

**Figure 3:**
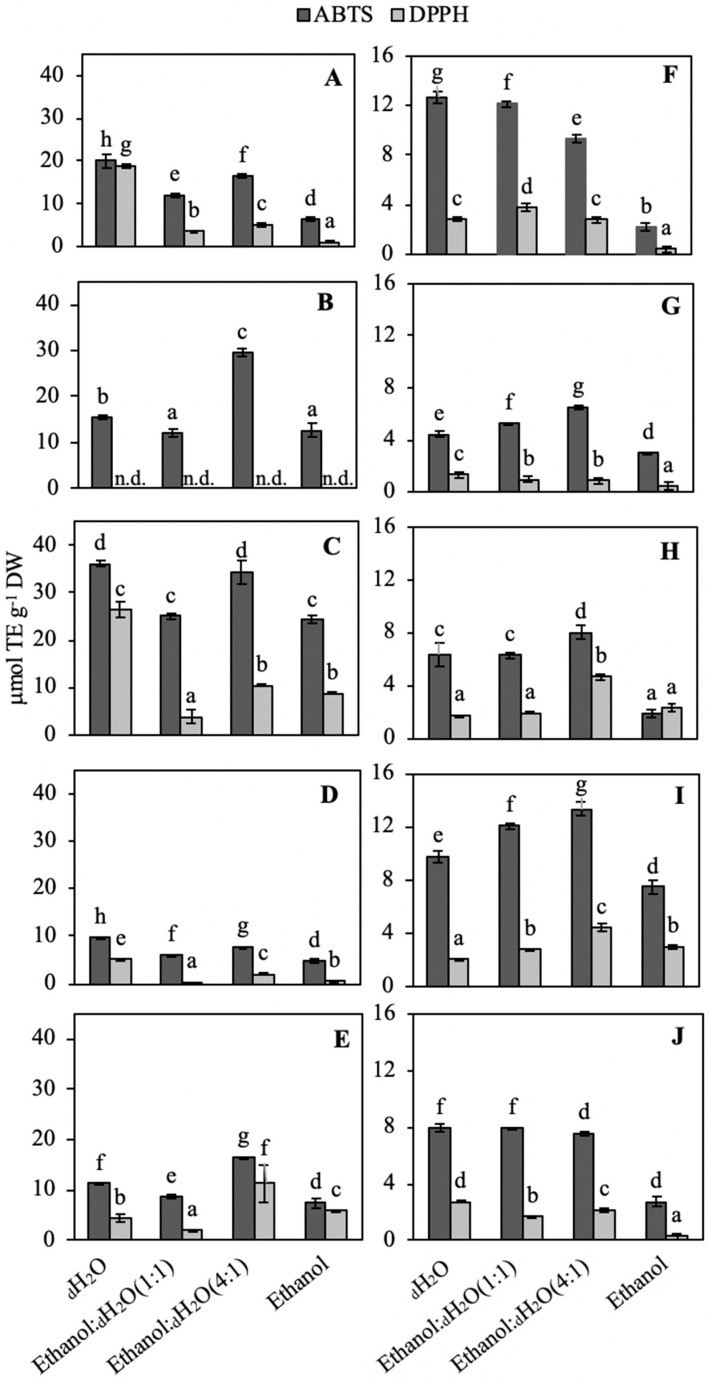
Antioxidant activity measured using the ABTS and DPPH assays (μmol TE g^−1^ DW) of the different cyanobacteria and red algal species extracted in different solvents (_d_H_2_O, ethanol:_d_H_2_O (1:1), ethanol:_d_H_2_O (4:1) and ethanol). Data are expressed as average ± standard deviation (SD) (*n* = 3). (**A**) *Nostoc commune*., (**B**) *Anabaena* sp., (**C**) *Scytonema* sp., (**D**) *Calothrix scopulorum*, (**E**) *Lyngbya* sp., (**F**) *Porphyra umbilicalis*, (**G**) *Gracilariopsis longissima*, (**H**) *Gelidium corneun*, (**I**) *Osmundea pinnatifida*, (**J**) *Ceramium rubrum*. Different letters indicate significant differences among solvents and between methods for each specie (ANOVA, *p* < 0.05, SNK test).

Results of the antioxidant activity are shown in Figure 3. In general, cyanobacteria showed a higher antioxidant activity compared to red macroalgae, reaching values close to 35 μmol TE g^−1^ DW (Figure 3A–E), whereas maximal values of red macroalgae were around 15 μmol TE g^−1^ DW (Figure 3F–J). In all cases, significant differences (*p* < 0.05) were found motivated by the interaction between solvents and extraction procedures (Table S1). In most of the cases, the ABTS assay reached higher values than the DPPH assay, and among solvents, different responses were observed. In cyanobacteria, *Scytonema* sp. presented the highest activity with the ABTS assay (specially using _d_H_2_O and ethanol:_d_H_2_O (4:1) as solvent; approx. 35 μmol TE^−1^ DW). However, with the DPPH method, this species only showed a high value with the aqueous solvent (25 μmol TE g^−1^ DW) (Figure 3C). *Anabaena* sp. showed higher values with the ABTS assay (ethanol:_d_H_2_O (4:1): 30 μmol TE g^−1^ DW), but no antioxidant response was detected when DPPH was used (Figure 3B). *N. commune* presented the highest antioxidant activity in aqueous solvent (in both ABTS and DPPH assays: 20 μmol TE g^−1^ DW) (Figure 3A). *Lyngbya* sp. reached the higher values in ethanol:_d_H_2_O (4:1) solvent (for ABTS: 18 mmol TE g^−1^ DW; and for DPPH: 12 mmol TE g^−1^ DW) (Figure 3E). *C. scopulorum*. was the cyanobacteria with the lowest antioxidant activity (Figure 3D).

Taking the red algae into account, the highest antioxidant values with the ABTS assay were observed in *O. pinnatifuda* and *P. umbilicalis*. In *O. pinnatifida*, the highest value was obtained using ethanol:_d_H_2_O (4:1) as solvent (14 μmol TE g^−1^ DW) (Figure 3I), whereas in *P. umbilicalis*, it was reached with the aqueous solvent (12.5 μmol TE g^−1^ DW) (Figure 3F). The highest values with the DPPH method were observed in *G. corneun* and *O. pinnatifida*, both using ethanol:_d_H_2_O (4:1) as solvent (approx. 4 μmol g^−1^ DW) (Figure 3H,I).

### 2.4. Photoprotective Capacity

**Figure 4:**
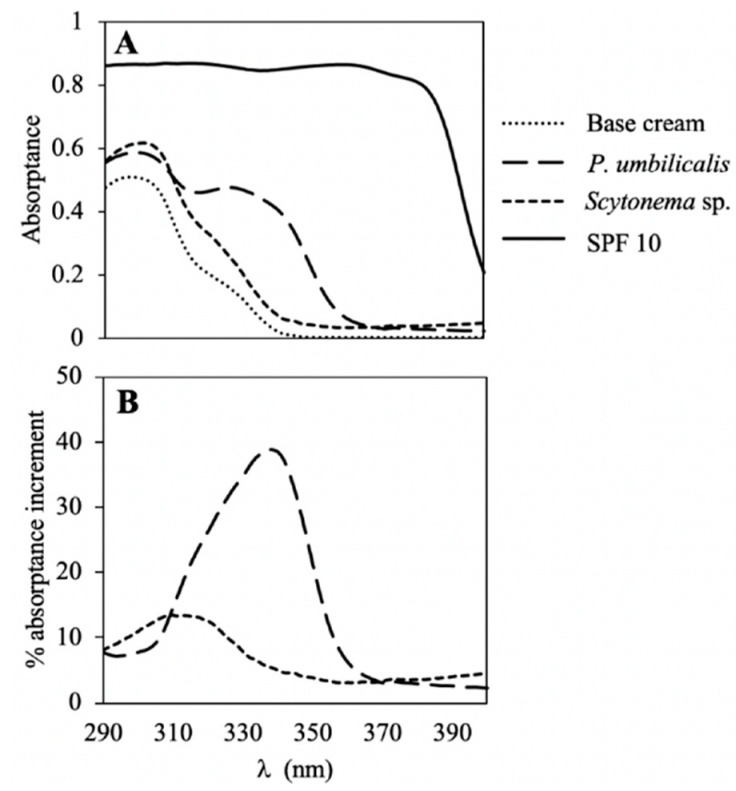
Photoprotection properties of creams with cyanobacteria and red alga extracts. (**A**) Absorptance of the different creams containing algae extracts (*P. umbilicalis* and *Scytonema* sp.), as well as the base cream and a control sunscreen with SPF 10. (**B**) The absorption increments due to the two extracts in comparison with the base cream.

The absorptions of the UV radiation by the creams including algae extracts are shown in Figure 4A. The capacity to protect against this type of radiation measured through two different indices is shown in Table 4. All creams including algal extracts increased the photoprotection when compared with the base cream (Figure 4A,B). In most of the cases, significant differences (*p* < 0.05) were observed when comparing the different creams containing algal extracts (Table S1).

In the cream with *P. umbilicalis* extract, the highest absorptance increase was observed in the UV-A region. Between 335 and 340 nm, the absorptance increased by close to 40% in comparison with the base cream. On the other hand, the cream with *Scytonema* sp. extract presented a highest absorption increase in the UV-B region. Between 309 and 312 nm, the absorptance increased by 15% in comparison with the base cream (Figure 4B).

Considering the calculated indexes, the cream with red algae extract reached an SPF of 2.1, which corresponded with 50% of the ESAR, followed by the protection against elastosis (1.5) and UVAPF (1.4) with an ESAR of 24 and 23%, respectively. Using the cyanobacteria extract, the SPF was similar to that obtained with red macroalgae, 1.9 and an ESAR of 47%, whereas the protection against UV-A rays was lower.

### 2.5. Correlations and Principal Component Analyses

Positive correlations (Table S2 and S3) (*p* < 0.05) were observed between antioxidant activity, mainly ABTS, and different molecules. In cyanobacteria, a high correlation was observed between the ABTS assay (in all solvents) and phenols content (in all solvents), e.g., for ABTS _d_H_2_O vs. phenols _d_H_2_O the r was 0.983. ABTS results for aqueous and hydroethanolic extracts (_d_H_2_O and ethanol:_d_H_2_O (1:1)) also showed positive correlation with polar molecules such as carbonated compounds (*r* = 0.7), soluble proteins (*r* = 0.6) and phycocyanin (*r* = 0.6). On the other hand, scytonemin showed positive correlations with ABTS and DPPH results (mainly with DPPH in ethanol, *r* = 0.977). DPPH also presented a positive correlation with phenols content.

In red algae, positive correlations were also obtained between ABTS and phenols content, e.g., ABTS vs. phenols in aqueous solvents reached a correlation coefficient r equal to 0.951. However, DPPH results did not show positive correlations with phenols content. The ABTS values obtained for aqueous solvents also showed a positive correlation with nitrogenous compounds: total nitrogen (*r* = 0.514), total proteins (*r* = 0.514), soluble proteins (*r* = 0.696), phycoerythrin (*r* = 0.576) and MAAs (*r* = 0.809).

**Figure 5:**
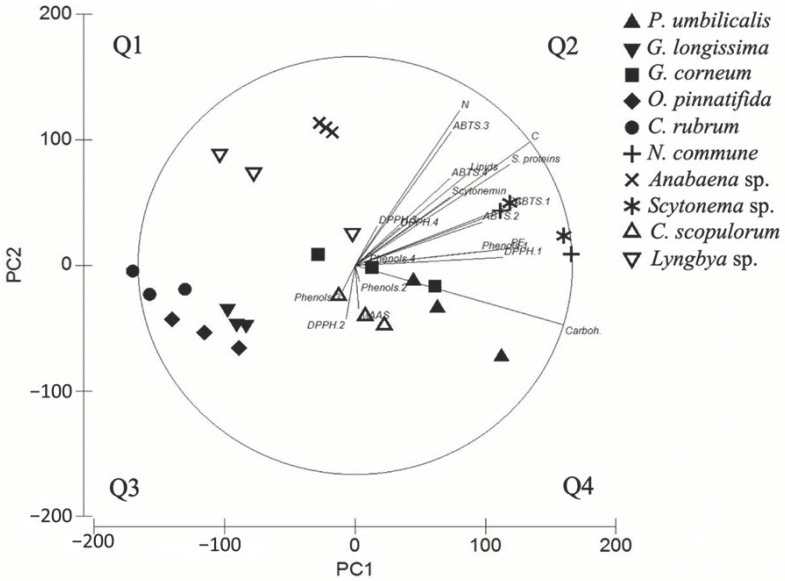
Results of the PCA applied to biochemical composition and antioxidant activities. Q1, Q2, Q3 and Q4 correspond to the different quadrants in the graph. Graphic codes represent the dependent variables as the following. ABTS.1: ABTS assay in _d_H_2_O extraction; ABTS.2: ABTS assay in ethanol: _d_H_2_O (1:1) extraction; ABTS.3: ABTS assay in ethanol:_d_H_2_O (4:1); ABTS.4: ABTS assay in ethanol extraction; DPPH.1: DPPH assay in _d_H_2_O extraction; DPPH.2: DPPH assay in ethanol:_d_H_2_O (1:1); DPPH.3: DPPH assay in ethanol:_d_H_2_O (4:1); DPPH.4: DPPH assay in ethanol extraction; C: total carbon content; N: total nitrogen content; S. proteins: soluble proteins; Carboh.: carbohydrates; Phenols.1: phenols content in _d_H_2_O extraction; Phenols.2: phenols content in ethanol:_d_H_2_O (1:1) extraction; Phenols.3: phenols content in ethanol:_d_H_2_O (4:1); Phenols.4: phenols content in ethanol extraction; PE: phycoerythrin; PC: phycocyanin; MAAs: mycosporine-like amino acids.

The results of the PCA (Figure 5) performed with all biochemical analysis and antioxidant activities showed that two principal components explained 96.8% of the variation. The first principal component (PC1) explained 79.1%, whereas the second (PC2) represented 17.7% of the total data variation. Based on the PCA graph and the correlations obtained between all variables and the different principal components (PC-axes) (Table S4), the PC1 was related with the polarity of the molecules, aqueous compounds (such carbohydrates, soluble proteins, phycobiliproteins and antioxidant activity obtained in aqueous solvents) being more correlated with this axis. The variables that most influence this axis are carbohydrates (quadrant 4 of Figure 5) with a correlation index of 0.959, followed by soluble proteins (0.711) and phycobiliproteins (PE and PC, 0.709 and 0.604), respectively, and antioxidant activities under more polar solvents (ABTS.1, ABTS.2 and DPPH.1) with correlation indexes of 0.726, 0.583 and 0.680, respectively (Table S4). The PC1 axis also divided some species in different groups: (1) three red macroalgae (*C. rubrum*, *O. pinnatifida* and *G. longissima*) grouped in quadrant 3 (Figure 5), (2) the other two red macroalgae and one cyanobacteria (*G. corneum*, *P. umbilicalis and C. scopulorum*) grouped in the central area of the axis mainly associated with quadrant 4 and (3) two cyanobacteria (*N. commune* and *Scytonema* sp.) grouped in quadrant 2. *N. commune* and *Scytonema* sp. are the species more associated with this axis, appearing in the first quadrant (Figure 5). In relation to the PC2 axis, the variable with the highest correlation is the N content with a correlation index of 0.742; other variables that can explain this axis are ABTS.3 and total carbon (0.640 and 0.590, respectively) (Table S4). This axis only separated clearly one cyanobacteria, *Anabaena* sp.

## 3. Discussion

### 3.1. Biochemical Composition

Algae contain several molecules in their composition with high interest due to their bioactivities. These compounds are normally divided into carbonated and nitrogenated ones. In general, the internal carbon (C) and nitrogen (N) contents and the C:N ratio can be physiological indicators, showing if algae are living in optimal conditions or under nitrogen limitation. In this study, the N content was lower in macroalgae than that in cyanobacteria, probably because the first ones were collected from their natural habitat, whereas the cyanobacteria biomasses were obtained by cultures under N-enriched supply and controlled growth conditions. The N content obtained in this study varied between 2.5 and 4.5% in red macroalgae and between 3.5 and 8.5% in cyanobacteria. Similar results were obtained by other authors in collected macroalgae [36,37]. In microalgae and cyanobacteria, the N percentage can also vary depending on the phase of the culture [38]. The optimal C:N ratio was described as 10 [39], whereas a higher proportion would indicate N limitation. In this study, all species showed a C:N ratio less than or equal to 10, indicating that the species used in this study were not N-limited.

All of the species evaluated in our study presented a high content of total internal carbon, i.e., most species values were higher than 30% of DW, similar to the average C content obtained in algae [40]. Considering C compounds, polysaccharides have acquired a great importance (in this work only the total C and total carbohydrates content were measured, the polysaccharides were not identified or quantified). Red algae present wall polysaccharides such as agars or carrageenans that are used as gelling and stabilizing agents [41,42] and could be used as excipients or functional ingredients in cosmetic formulations [15]. Polysaccharides have also been studied for their antitumoral, antioxidant and immunological activities [43,44,45]. These compounds in cyanobacteria have been less studied, although some authors reported the production of exopolysacharides (EPS) by some cyanobacteria with different bioactivities [46,47,48].

Macroalgae normally present low lipid content (as observed in this study, <2% DW) [49]. Cyanobacteria also showed low values, whereas other authors obtained higher values. In spite of the low values, these organisms can produce high value fatty acids [50]. A possible cosmetic application of lipids in cosmetic products is the avoidance of skin dehydration [15].

Phenolic compounds are C compounds with a role as natural antioxidants, as it has been shown by several authors [51,52]. More recently, they have also been studied for their photoprotective applications [53,54,55]. Brown algae are the main producers of these compounds, however, cyanobacteria and red algae can also produce phenols, although in less quantity [37]. In this work, it was possible to find an optimization of phenolics extraction when different solvents were utilized. In our case, water was the best solvent for their extraction, similarly to other studies [52,56]. In red algae, the highest content was observed in *P. umbilicalis* and *O. pinnatifida* (approx. 14 mg g^−1^ DW). High levels of polyphenols (25 mg GAE (gallic acid equivalent) g^−1^ DW) were also observed in *Porphyra tenera* [57], applying similar extraction conditions. Zubia et al. obtained similar results in species from the Gracilariales family [58]. Less information was found in relation to the phenolic content in cyanobacteria. Li et al. obtained approx. 2.5 mg GAE g^−1^ DW in aqueous extracts of *Anabaena flos-aquae* and *Nostoc sllipsosporum* [59]. Singh et al. obtained higher phenols contents in *Anabena* sp., *Nostoc* sp. and *Lyngbya* sp. (60, 40 and 92 mg GAE g^−1^ FW, respectively) [60].

On N compounds, proteins or derivates (peptides or aminoacids) show antioxidant or immune-stimulant properties and can also confer moisture retention in the skin [61]. Total and soluble proteins were in general higher in three species of cyanobacteria (*N. commune, Anabaena* sp. and *Scytonema* sp). The soluble proteins represent about 20–25% of total proteins except in *Anabaena* sp. (9.9%) and *C. scopulorum* (5%), thus, the level of total protein in *Anabaena* sp. is much higher than the other species (40.6%). This can be explained by the contribution of structural proteins, which could be higher than in the other cyanobacteria. It is also necessary to remark that total protein was estimated from a nitrogen to protein conversion factor; it would be possible that the applied factor overestimated in the case of *Anabaena* sp. due to its amino acid profile. Among the red algae, only *P. umbilicalis* presents high values, close to most cyanobacteria. *P. umbilicalis* is a species known for its used as human food (e.g., nori) [62]. *Nostoc* sp. and *Anabena* sp. also present a high nutritional value and are used as food in some countries [63].

Phycobiliproteins are N molecules that act as accessory pigments responsible for the light harvesting in both group of organisms analyzed. Cyanobacteria presented a high content of biliproteins, mainly phycocyanin. The percentage of biliproteins related to soluble proteins was higher than 30% in *N. commune* (53.8%), *Scytonema* sp. (37%) and *Lyngbya* sp. (31.8%), whereas the other two species presented much lower percentages, e.g., *C. scopulorum* (18.75%) and *Anabaena* sp. (7.5%). Other authors obtained similar and higher values of biliproteins than those obtained in this study [64,65,66]. In red algae, the percentage of biliproteins related to soluble proteins was higher in *P. umbilicalis* (13.3%) and *G. corneum* (13.6%), in comparison with the other species, e.g., *G. longissima* (10%), *C. rubrum* (5.2%) and *O. pinnatifida* (3.7%). The level of biliproteins was lower than that obtained in collected algae by other authors [67,68]. Biliprotein concentrations can vary depending on environmental factors like irradiance, light quality or nutrients [69,70,71]. The extraction method can also influence the content, e.g., a great number of authors used the freezing and thawing method [72].

Scytonemin, one of the main photoprotective molecules found in cyanobacteria, is a lipophylic and yellow-brown pigment with the capacity to absorb UVR, mainly in the UV-A-violet-blue region with a maximum absorption at 384 nm [73]. This pigment is only presented in the extracellular sheaths of some cyanobacteria [74,75]. All the cyanobacteria tested showed scytonemin in their composition, in contrast to Mushir et al., who did not observe this pigment in *Lyngbya* sp. or *Calothrix brevissema* [76]. Other authors also observed scytonemin in the same species tested in this study [77,78,79,80]. In this study the highest level of scytonemin was reached in *Scytonema* sp. followed by *Lyngbya* sp., showing similar amounts as those reported in other studies [76,81]. These results can be compared with the UV spectra, in which the scytonemin peaks (380 nm) were visible only in the most polar solvents (ethanol:_d_H_2_O (4:1) and pure ethanol) and were more prominent in *Scytonema* sp., *Lyngbya* sp. and *N. commune*. The culture or environment conditions can vary the scytonemin content, e.g., nitrogen deficiency can increase scytonemin [82].

Mycosporine-like amino acids (MAAs) are secondary N metabolites also implicated in the protection against the UV radiation and mainly reported in the two groups of organisms tested in this study [83,84]. Most of the species analyzed presented MAAs. In the case of cyanobacteria, only *Scytonema* sp. and *Lyngbya* sp. showed quantified peaks in the HPLC. Other authors also observed MAAs in different species of the cyanobacteria genera tested [85,86,87]. Sinha et al. also identified an excreted UV absorbing compound in the culture media of *Scytonema* sp., although in our study the medium was not analyzed [88]. In red algae, different authors observed the highest content of MAAs in the order Bangiales in which *P. umbilicalis* is included [89]. The concentrations of MAAs obtained in this study were similar to those observed in the bibliography [89,90,91,92,93,94].

There are different types of MAAs that differ in the chemical formula and the λ_max_ [84,95]. *Scytonema* sp. was the unique species that presented an MAA with a λ_max_ in the UV-B region. In this study, it was identified as mycosporine-glutaminol for the first time in *Scytonema* sp., although Rastogi et al. observed mycosporine-glycine and the unknown MAA-334 in this species [72]. Rastogi and Incharoensakdi also observed palythine and asterina-330 in *Lyngbya* sp. [78]. In red algae, similar patterns were observed by other authors. In *P. umbilicalis*, different MAAs were detected, the predominant being porphyra-334, as observed in *Porphyra* spp. or *Pyropia* spp. [92,96]. In *G. longissima*, only shinorine was detected, in contrast to other authors who in the same specie observed more types of MAAs [96,97,98]. Briani et al. also studied species of the genus *Gelidium* obtaining similar results, high content of asterina-330 and shinorine [92]. In the genera *Laurencia* and *Osmundea*, a high content of asterina-330 was also observed [92,99]. In *C. rubrum*, shinorine was also detected, whereas other authors also observed different MAAs in similar species [92,96]. The similarities in the qualitative composition of MAAs found by different authors in similar species suggest that the composition of the main MAAs can be conservative among species, although the percentage of each one can be modified by some environmental factors, such as the presence of UV radiation or a high intensity of solar radiation [99,100].

The different composition of MAAs among species is relevant for cosmeceutical applications, since not all MAAs absorb in the same wavelengths or present the same antioxidant capacity [101,102,103]. The MAAs concentration/composition can be compared with the UV absorption spectra. A peak in the UV-B region (310–312 nm) was only observed in *Scytonema* sp., whereas the other species present peaks with maximum absorbance between 330–335 nm depending on the predominant MAAs. The highest peak was observed in *P. umbilicalis*, which means the highest content in MAAs. The UV spectra agreed with the HPLC results. MAAs peaks were similar in most of the solvents, except in pure ethanol. MAAs are water soluble molecules, but these results suggest that only 20% of water can extract approximately the same as 100% water.

### 3.2. Bioactivities (Antioxidant and Photoprotective) and its Relation with Biochemical Composition

Most of the above discussed molecules can act as antioxidant compounds. The general antioxidant activity measured through two different methods based on the free radical scavenging activity was higher in cyanobacteria than in red macroalgae. In cyanobacteria, the highest antioxidant activity was obtained in the order Nostocales (the group of *Scytonema* sp., *Anabaena* sp. and *Nostoc* sp.) as has been reported by other authors [60,104,105,106]. Considering the red macroalgae, the highest antioxidant activity was reached in the species *O. pinnatifida*, *P. umbilicalis* and *C. rubrum*. Other authors also found a high antioxidant activity in the order Ceramiales (where *O. pinnatifida* and *C. rubrum* belong), Gracilariales and Bangiales [37,58,97,107].

The differences among solvents used for the extractions can depend on the molecules extracted in each solvent, i.e., proteins, carbohydrates, MAAs and phycobiliproteins are water soluble, whereas chlorophylls, carotenoids and lipids are soluble inorganic solvents like ethanol, methanol or acetone. Many authors also studied the influence of solvents in antioxidant activity, and different results were observed depending on the species and method applied [97,108,109]. In this work only aqueous and ethanolic solvents were tested due to the non-toxicity needed for cosmetic purposes. The method used also influenced the results. In this study, most of the cases showed the highest antioxidant activity using the ABTS assay [109]. ABTS is dissolved in _d_H_2_O but can also be dissolved in water based solvents such as ethanol/methanol:water (1:1) [110], whereas DPPH can only be dissolved in organic media, limiting the measure of hydrophilic antioxidants. Some interferences can appear when the absorbance is measured at low wavelength as in the DPPH method [111], and the different molecules presented in the extracts can also influence the results [112].

In general, positive correlations were obtained between the antioxidant activity and the different analyzed molecules. Biliproteins showed a positive correlation with the antioxidant activity [113,114], and two of our tested cyanobacteria presented strong association between them (*Scytonema* sp. and *N. commune*, Figure 5). In addition, the proportion of biliproteins related to soluble proteins is very high in the last cited species, 37.0% and 53.8%, respectively, thus providing high content of biliproteins for cosmeceutical applications. In the case of cyanobacteria, scytonemin also showed positive correlations with antioxidant activity, as observed by Matsui et al., who showed the radical-scavenging activity by this molecule [115]. Polyphenols also presented positive correlations with the antioxidant activity in both groups of organisms tested, as has been observed by several studies [116,117]. Finally, MAAs only showed correlation with the antioxidant activity in red algae, as has been previously reported [101,102,118].

The PCA showed that the PC1 axis explained 79.1% and it can be explained by the polarity of the molecules analyzed, with more positive relation with polar extractions (water), whereas the PC2 represents 17.7% of the obtained data and it mostly related to the total nitrogen content. PC1 is mainly related to C content, followed by soluble proteins. The different groups obtained by the PC1 axis seemed to be separated by their content water-soluble compounds (mainly carbonated compounds), the first group, presented in quadrant 3 (negative part of the PC1 axis), being the species with the lowest content and the third group, presented in quadrant 1 (positive part of the axis), the species with the highest content. However, in the PC2 axis only *Anabaena* sp. was clearly separated due to its high N content. *Lyngbya* sp. also appeared near to this axis but presented a high variability. Scytonemin presented low and similar correlations with both axes; the fact that red macroalgae did not present this molecule could interfere in the results. Lipids also presented similar correlations with both axes, probably due to their carbonated and lypophylic character. On the other hand, some analyzed molecules did not follow the pattern described before: MAAs should be correlated with the PC2 axis, although these molecules showed a low correlation with both main axes. As for scytonemin, the fact that not all species presented MAAs could interfere in the results. Phenols content only showed correlation with one of the axes (PC1) when it was extracted in water; the other extraction also showed low correlation with both axes. Finally, the species of red algae more taxonomically related are also grouped in the PCA analysis, e.g., the Florideophyceae *O. pinnatifida*, *C. rubrum* (Ceramiales order) and *G. longissima* (Gracilariales order) formed a group, and the other group is formed by *P. umbilicalis* (Bangiales order) and *G. corneum*, a Florideophyceae algae of Gelidiales order but closer in the phylogenia to Bangiales than the other studied Florideophyceae algae [119].

The photoprotective capacity was measured in those species that showed a high content of interesting molecules and antioxidant activities (*P. umbilicalis* and *Scytonema* sp.). Hydroethanolic (1:1) solvent was selected for this analysis because this solvent might facilitate the extraction of combined molecules, water and organic soluble.

In Figure 4, the peak observed in the cream with *P. umbilicalis*, in the UV-AII region (320–340 nm), can be related to the MAAs content, increasing more the photoprotection against the effects mostly related to UVA radiation, e.g., using the extract of *P. umbilicalis*, the ESAR_PPD_ increased 500% and the BEPF_PPD_ 38%, with respect to the base cream. In the case of the cream with *Scytonema* sp., the high absorptance observed in the UVB region could be due to its MAAs content, which absorbed in the UVB region (mycosporine-glutaminol), instead of porphyra-334 (presence in *P. umbilicalis*), which absorbed in the UVA region.

The increase of the protection factors (SPF, UVAPF and BEPFs) in comparison with the increase of the ESAR shows a hyperbolic response, as described Schneider et al., i.e., at the beginning of the curve, an increase in the ESAR is expressed in a very small increment in the protection factors values, i.e., creams with a SPF of 2 can absorb 50% of the UV radiation [35]. However, when the capacity to absorb the damage radiation reached 80%, the increase in the SPF was much higher, i.e., a SPF of 5 means that it absorbs 80% of the UVR, a SPF of 15 absorbs around 90% and a SPF of 30 absorbs around 95%. Results obtained in the present work are at the beginning of the curve, reaching 50% of the ESAR.

Many authors have studied the potential of MAAs as UV-filtering compounds [35,93,98,103], but few articles have reported the inclusion of these molecules as UV-absorbing compounds in lotions. De la Coba et al. studied different formulations with MAAs, adding around 5% of purified MAAs and obtained values of SPF that varied from 4.5 to 8.3 [34]. In our study, the % of MAAs included in the creams was much lower, varying from 0.003 to 0.026% due to the dilutions in the cream, explaining the reasons why we found lower values of SPF (around 2). Lawrence et al. found a high UV photoprotection in human keratinocites using a 0.3% palythine [103]. Other authors studied the protection of galenic formulations with MAAs in mouse skin, obtaining positive results [120,121]. MAAs are promising substances in the cosmetic field due to their high stability against UV radiation, pH or temperature [34,78,122], their antioxidant capacity [34,102] and other biological activities such as anti-inflammatory and inhibition of enzymes related to photoaging as collagenase [34,123,124]. In the market, there are some examples of natural antioxidants and photoprotectors based on red algae extracts, e.g., Helionori^®^ from Gelyma or RonaCare^®^ RenouMer from Merck, which contains MAAs and presents cosmetic properties. Another example of a natural product is Heliocare^®^, based on extracts of the fern *Polyplodium leucotomos,* enriched in polyphenols with antioxidant and photoprotective properties and known as Fernblock^®^ [125,126]. Thus, those species with highest level of polyphenols (12–22 mg g^−1^ DW), e.g., the cyanobacyteria *N.commune* and *Scytonema* sp and the red macroalgae *P.umbilicalis* and *O.pinnatifida*, could be used as sources of polyphenols for cosmetic products.

Finally, the inclusion of scytonemin in lotions to act as sunscreen has not been reported yet. However, the possibility to use it as photoprotector in cosmetic products have been discussed [127]. In our study, the scytonemin peak was not observed in the creams, probably due to the selected solvent that extracted less pigments in comparison with a less polar solvent such as ethanol or methanol. Scytonemin should be extracted in a solvent compatible with its application in cosmetic products (e.g., ethanol or combinations of water:ethanol) for a better application of this compound as photoprotector. This pigment has been less studied than MAAs, although some properties have been described. It is a stable molecule [78] with positive biological activities (antioxidant or anti-inflammatory) for the skin [115,128], which could be a good option as biological photoprotectors.

### 3.3. Future Perspectives

The potential application of these organisms as sources of commercial bioactive compounds requires high biomass availability and high biocompounds productivity. Red algae (specially *Porphyra* genus and *Gracilariales* or *Gigartinales* orders) have been cultured for centuries, mainly in Asian countries, for human consumption or for their agar or carrageenan content [62]. These species are normally produced in open sea water using ropes, although they can also be produced under integrated multitrophic aquaculture (IMTA). In this way, algae can use the nitrogen and phosphate coming from the fish pond effluents to produce high valuable compounds [129,130]. As mentioned before, N compounds such as phycobiliproteins or MAAs can be increased under high nutrients availability. On the other hand, cyanobacteria can be isolated and cultured easily in liquid medium, with the possibility to scale up using raceways or thin-layer cascade systems [131]. Bioremediation is also an option for these species. Thereby, algal biomass with high bioactivity could be produced at lower cost and the environmental impact could also be reduced. Apart from the need of a big-scale culture, a high yield extraction, concentration and/or purification (using green techniques) of both interested molecules (MAAs and scytonemin) is a challenge for the natural cosmetic industry [132,133,134], increasing the perspective of utilization of these species for sunscreen formulations.

## 4. Conclusions

This study shows the usefulness of extracts of different species of cyanobacteria and red macroalgae as cosmeceuticals due to both their antioxidant and photoprotection capacity. Aqueous and hydroethanolic extracts of different species presented antioxidant activity that can be attributed to different molecules, mainly polyphenols, MAAs and pigments (biliproteins and scytonemin). Hydroethanolic extracts of *P. umbilicalis* and *Scytonema* sp. that contain MAAs could increase the photoprotection of sunscreens. The concentration or purification of the interested molecules is needed for better photoprotection. *Porphyra* spp. are good candidates for cosmetic applications due to their high content of MAAs and cultivation possibilities. In addition, *Scytonema* sp. can also contribute in the photoprotection of UV-B and UV-A mediated responses due to its MAAs (absorption in the UV-B region) and scytonemin (absorption in the UVA-I region) content. Thus, red algal and cyanobacteria extracts enriched in MAAs and scytonemin can be useful to design broad-band photoprotectors in both UV-A and UV-B regions of the spectra.

## 5. Materials and Methods

### 5.1. Biological Material

Ten species of both cyanobacteria and red macroalgae were employed in this study. Cyanobacterium clonal strains were provided by the Culture Collection at the Spanish Bank of Algae (Taliarte, Gran Canaria, Canary Islands) (Table 5). Cultures were scaled up to 1 L flasks, under controlled laboratory conditions (irradiance: 100 μmol photons m^−2^ s^−1^; photoperiod: 16:8 (L:D); temperature: 23 ± 2 °C) and continuous aeration supplied with CO_2_ pulse addition at a rate of 1 min every hour. Culture medium was BG11. Biomass samples were harvested and freeze-dried (6.5 L Labconco, Kansas, MO, USA) before carrying out the extractions. Red macroalgae were collected in coastal areas of Cadiz and Malaga in the Southern Iberian Peninsula (Table 5), transported to the laboratory in a portable fridge at 4 °C, washed and cleaned for removal of sediments and epiphytes, frozen at −40 °C and freeze-dried (Cryodos, Telstar, Barcelona, Spain). Criteria for the selection of these species were (1) biomass availability, both by collection in nature or culture under controlled conditions, and (2) the potential content of bioactive compounds with photoprotection capacity.

### 5.2. Preparation of Algal Extracts

Lyophilized samples were ground with a pestle and mortar and extracted in different solvents: distilled H_2_O (_d_H_2_O), ethanol:_d_H_2_O (1:1), ethanol:_d_H_2_O (4:1) and ethanol. Three hundred mg of dry weigh (DW) were homogenized in 10 mL of the solvent (concentration: 30 mg mL^−1^) and incubated in a thermal bath at 45 °C for 6 h. After this time, extracts were filtered and centrifuged (Sartorius 2-16PK, Sigma, Germany) at 2000× *g* for 15 min.

Absorbances or optical densities in the UV region (290 to 400 nm) of the different extracts were measured using a UV-visible spectrophotometer (UV Mini-1240, Shimadzu, Columbia, MD, USA). Extracts in the different solvents were used to compare the content of phenolic compounds and the antioxidant activity. Aqueous extracts were also used for the quantification of soluble proteins, phycobiliproteins and mycosporine-like amino acids (MAAs).

### 5.3. Biochemical Composition

#### 5.3.1. Total Carbon and Nitrogen

Total internal carbon and nitrogen contents were determined from dry biomass of red algae and cyanobacteria using a CNHS LECO-932 elemental analyzer (St. Joseph, MI, USA) in the Research Support Central Services (SCAI, University of Málaga, Málaga, Spain). Results were expressed as mg g^−1^ DW.

#### 5.3.2. Proteins

Total proteins were calculated by using the nitrogen to protein conversion factor, 4.92 for macroalgae [36] and 4.78 for cyanobacteria [38]. Soluble proteins were quantified according to the Bradford method [135]. Twenty μL of the aqueous extracts (item 2.2) were mixed with 780 μL of phosphate buffer (0.1 M, pH 6.5) and 200 μL of Biorad solution (Bio-rad, Feldkirchen, Germany). After 15 min of incubation at room temperature and darkness, absorbances were measured at 595 nm. Bovine serum albumin was used as standard. In both cases, results were expressed as mg g^−1^ DW.

#### 5.3.3. Carbohydrates

Carbohydrates were quantified according to the phenol-sulfuric acid method [136]. Five mg of DW were homogenized in 5 mL of 1 M H_2_SO_4_ and incubated for 1 h at 100 °C; after cool down, extracts were centrifuged (2000× *g*; 10 min). For the reaction, 1 mL of the supernatant was mixed with 1 mL of phenol 5%, incubated for 40 min at room temperature, and mixed with 5 mL of concentrated H_2_SO_4_. Absorbances were measured at 485 nm. Glucose was used as a standard and results were expressed as mg g^−1^ DW.

#### 5.3.4. Lipids

Total lipids were determined spectrophotometrically using the sulpho-phospho-vanillin (SPV) method [137]. Twenty mg of DW were homogenized in 1 mL of methanol. After centrifugation (2000× *g*, 10 min), 100 μL of the supernatant were mixed with 1 mL of H_2_SO_4_ and incubated for 10 min at 100 °C. After cool down, 2.5 mL of phospho-vainillin solution (0.6 g of vainillin, 100 mL of ethanol and 400 mL of H_3_PO_4_ 85%) were added to the mix, which was incubated for 15 min at environmental temperature and measured spectrophotometrically at 530 nm. Trioleine was used as standard and results were expressed as mg g^−1^ DW.

#### 5.3.5. Phycobiliproteins and Scytonemin

Aqueous extracts (item 2.2) were used for phycobiliproteins determination. In red algae, the quantification was made according to the formula described by Beer and Eshel, and in the case of cyanobacteria, the one proposed by Bennet and Bogorad [138,139]. 

For the extraction of scytonemin in cyanobacteria, 20 mg of DW was extracted in 1 mL of acetone. The quantification was made according to the formula described by García-Pichel and Castenholz, using the extinction coefficients reported by García-Pichel et al. [74,140].

Results of both compounds were expressed as mg g^−1^ DW.

#### 5.3.6. Phenolic Compounds

Phenolic compounds were determined from the different extracts using the Folin-Ciocalteu method [141] with some modifications. Briefly, 100 μL of the different extracts (item 2.2) were mixed with 700 μL of distilled water and 50 μL of Folin-Ciocalteu phenol reagent (Sigma-Aldrich, St. Louis, MO, USA). After vortexing, 150 μL of Na_2_CO_3_ 20% were added and samples were mixed again. The mixtures were incubated for 2 h at 4 °C in darkness. Absorbances were measured at 760 nm. Phloroglucinol was used as standard and results were expressed as mg g^−1^ DW.

#### 5.3.7. Mycosporine-Like Amino Acids (MAAs)

MAAs were determined using HPLC (high performance liquid chromatography) according to Korbee-Peinado et al., with some modifications of Chaves-Peña et al. [96,142]. Seven hundred microliters of the aqueous extracts (item 2.2) were dried under vacuum. The dry extract was re-suspended in 700 μL of methanol, filtered through a 0.2 μm membrane and analyzed using HPLC (Waters 600 HPLC, Waters Cromatografía, Barcelona, Spain). MAAs were detected using a Luna C8 column (Phenomenex, Aschaffenburg, Germany), applying an isocratic flow of 0.5 mL min^−1^ and a mobile phase of 1.5% methanol and 0.15% acetic acid in ultrapure water. The detection was made using a photodiode array (PDA) detector at 310 and 330 nm. Secondary standards were used for the identification of MAAs, and the quantification was performed using the molar extinction coefficients (ε) of the different MAAs [143]. Results were expressed as mg g^−1^ DW.

The MAAs were also identified via positive electrospray ionization mass spectrometry (ESI-MS) (Orbitrap Q-Exactive, Thermo Scientific S.L., Bremen, Germany) in the Research Support Central Services (SCAI, University of Malaga, Spain).

### 5.4. Antioxidant Activity (ABTS and DPPH)

The antioxidant activity of the different extracts was evaluated through two different methods based on the free radical scavenging activity.

The ABTS assay was performed according to Re et al. [144] with some modifications. The ABTS radical cations (ABTS^+•^) were generated via a reaction of 7 mM ABTS (2,2′-azino-bis(3-ethylbenzothiazoline-6-sulfonic acid) and 2.45 mM K_2_S_2_O_8_ in phosphate buffer (0.1M, pH:6.5). This reaction was stored for 12–16 h at room temperature to ensure the complete formation of the radical. ABTS^+•^ solution was diluted with phosphate buffer until the absorbance at 727 nm was around 0.75 ± 0.05. For the reaction, 50 μL of the different extracts (item 2.2) were mixed with 950 μL of the diluted ABTS^+•^. The mixture was incubated for 8 min at room temperature and darkness, and absorbances were measured at 727 nm.

The DPPH assay was made according to Brand-Williams et al. [145] with some modifications. For the reaction, 200 μL of the different extracts (item 2.2) were mixed with 1 mL of the DPPH^•^ (2,2-diphenyl-1-picrylhydrazyl) solution (0.06 mM of DPPH in methanol 80%). After 30 min of incubation at room temperature and darkness, absorbances were measured at 517 nm.

For both methods, a standard solution of Trolox (6-hydroxy-2,5,7,8-tetramethylchroman-2-carboxylic acid) was used as reference and the results were expressed as μmol TE (Trolox equivalent) g^−1^ DW.

### 5.5. Photoprotection Capacity

The photoprotection capacities were measured in vitro in those species in which the highest content of interest molecules was observed. The selected species were one cyanobacterium (*Scytonema* sp.) and one red macroalga (*P. umbilicalis)*. Hydroethanolic (1:1) extracts were used for this analysis. Extracts (item 2.2) were concentrated under vacuum in a rotary evaporator (Büchi R-210, Switzerland) from 50 to 200 mg mL^−1^. Extracts were incorporated in an oil-water cream (25% w/w). One hundred milligrams of the creams were spread in a polymethylmethacrylate (PMMA) plate (Schönberg, Hamburg, Germany), with a surface of 50 mm × 50 mm and a roughness (Ra) in the range of 4.5–5.2 μm. Creams were distributed on the plates with gloved fingertips for 30 s (approx.); after that, plates were allowed to dry for 15 min in darkness. Transmission through plates was measured using a spectrophotometer (UV-2700i, Shimadzu, Duisburg, Germany) with an integrating sphere. The sun protection factor (SPF), UVA protection factor (UVAPF) and biological effective protection factors (BEPFs) considering other action spectra (elastosis and photoaging) were calculated using the following equation, as De la Coba et al. (2019) [34]:

$$SPF,\;UVAPF,\;BEPFs=\frac{\sum_{290}^{400}\mathrm{Act}.\mathrm{Sp}\left(\lambda \right)*E\left(\lambda \right)}{\sum_{290}^{400}T\left(\lambda \right)*\mathrm{Act}.\mathrm{Sp}\left(\lambda \right)*E\left(\lambda \right)}$$

In addition, the effective solar absorpted radiation (ESAR) against different action spectra was determined as described by Schneider et al. (2020) [35]:

$$\%ESAR=\frac{\sum_{290}^{400}A\left(\lambda \right)*\mathrm{Act}.\mathrm{Sp}\left(\lambda \right)*E\left(\lambda \right)}{\sum_{290}^{400}\mathrm{Act}.\mathrm{Sp}\left(\lambda \right)*E\left(\lambda \right)}$$

**Figure 6:**
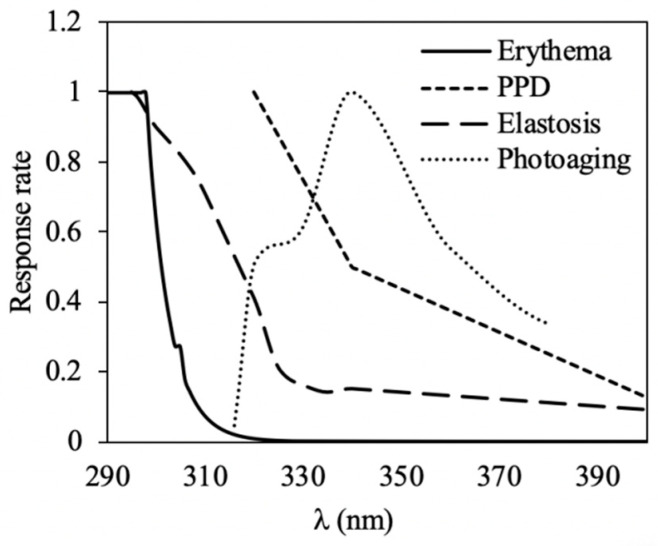
Normalized action spectra responses of different harmful effects related to skin damages driven by UV radiation and used in this study for the determination of the photoprotection capacity.

In both formulae,

Act.Sp (λ) = action spectra (Figure 6);
E (λ) = spectral irradiance of a sunny midday in a summer day (June) in Malaga (W m^−2^);
T (λ) = transmittance values at each wavelength (0–1);
A (λ) = absorptance values at each wavelength (0–1).

The different relative action spectra used in this study are shown in Figure 6. A commercial cream with SPF 10 that contains different chemical filters (butyl methoxydibenzoylmethane, ethylhexyl methoxycinnamate, methylene bis-benzotriazolyltetramethylbutylphenol and octocrylene) and titanium dioxide as a physical filter was used as positive control. In this case, 32 mg were spread on the plate according to COLIPA (2011) and Pissavini et al. (2018) [29,30].

### 5.6. Statistical Analyses

The software STATISTICA (V.7) was used for the statistical analysis. Analyses of variance (ANOVA) were done to compare data obtained. Two-way ANOVA were performed to detect significant differences in the antioxidant activity values, comparing solvents and methods. One-way ANOVA were used to evaluate species effects in all the biochemical compounds and antioxidant activity among species (cyanobacteria and red macroalgae were compared separately), the extraction of phenolic compound in each solvent and the photoprotection capacity. A Student-Newman-Keuls (SNK) post-hoc test was performed to compare the data after significant differences in the ANOVA. Homogeneities of variances were tested using the Cochran test and via visual inspection of the residuals. Correlations among dependent variable data were calculated using Pearson’s correlation analysis.

The software PRIMER 6 v.6.1.13 & PERMANOVA+ v.1.0.3 was used for the principal component analysis (PCA), performed to detect patterns among the biochemical composition and antioxidant activities of the different species. Data were correlated with each PC-axis using Pearson’s correlation coefficient.

## Figures and Tables

**Table 1:** Total internal carbon and nitrogen, proteins, carbohydrates and lipid contents (mg g^−1^ DW) and the C:N ratio of red algae and cyanobacteria expressed as average ± standard deviation (SD) (*n* = 3). Different letters indicate significant differences among species of cyanobacteria and red algae (ANOVA, *p* < 0.05, SNK test).

Species	C	N	C:N	Total Proteins	Soluble Proteins	Carbohydrates	Lipids
*N. commune*.	420.1 ± 0.2 ^d^	59.3 ± 0.3 ^c^	7.09 ± 0.04 ^c^	283.4 ± 1.3 ^c^	70.6 ± 1.7 ^e^	407.8 ± 64.8 ^d^	21.1 ± 0.3 ^c^
*Anabaena* sp.	406.8 ± 0.6 ^c^	85.1 ± 0.2 ^e^	4.78 ± 002 ^a^	406.9 ± 1.0 ^e^	40.4 ± 0.2 ^b^	185.4 ± 18.9 ^a^	15.5 ± 0.8 ^a^
*Scytonema* sp.	426.9 ± 0.8 ^e^	61.8 ± 0.2 ^d^	6.91 ± 0.02 ^b^	295.3 ± 1.1 ^d^	62.0 ± 0.5 ^d^	196.4 ± 63.6 ^a^	17.9 ± 0.2 ^b^
*C. scopulorum*	313.3 ± 1.2 ^a^	34.6 ± 0.3 ^a^	9.06 ± 0.02 ^e^	165.4 ±1.2 ^a^	8.3 ± 0.1 ^a^	285.6 ± 21.3 ^b^	19.0 ± 1.1 ^b^
*Lyngbya* sp.	352.5 ± 0.9 ^b^	42.8 ± 0.3 ^b^	8.23 ± 0.03 ^d^	204.8 ± 1.5 ^b^	44.3 ± 2.0 ^c^	334.1 ± 40.3 ^c^	18.2 ± 0.8 ^b^
*P. umbilicalis*	340.7 ± 11.5 ^c^	44.5 ± 2.9 ^d^	7.65 ± 0.24 ^b^	219.1 ± 14.3 ^d^	33.9 ±1.8 ^e^	318.5 ± 19.2 ^d^	5.7 ± 0.1 ^b^
*G. longissima*	255.8 ± 5.5 ^b^	29.7 ± 3.0 ^b^	8.66 ± 1.06 ^d^	146.3 ± 14.8 ^b^	7.8 ± 0.2 ^b^	206.5 ± 12.0 ^b^	4.8 ± 0.1 ^a^
*G. corneum*	351.2 ± 19.4 ^c^	33.2 ± 3.0 ^c^	10.58 ± 0.38 ^c^	163.6 ± 14.8 ^c^	11.2 ± 0.2 ^c^	274.8 ± 44.9 ^c^	4.8 ± 0.3 ^a^
*O. pinnatifida*	231.2 ± 5.6 ^a^	25.1 ± 1.7 ^a^	9.23 ± 0.40 ^c^	123.4 ± 8.4 ^a^	5.4 ± 0.5 ^a^	192.1 ± 27.9 ^b^	7.5 ± 0.3 ^c^
*C. rubrum*	239.4 ± 13.4 ^a^	36.0 ± 2.4 ^c^	6.65 ± 0.07 ^a^	177.1 ± 11.8 ^c^	19.4 ± 1.2 ^d^	139.5 ± 29.8 ^a^	5.5 ± 0.1 ^b^

**Table 2:** Phycobiliproteins content of cyanobacteria and red algae species and scytonemin content in cyanobacteria species (mg g^−1^ DW). Pigments were expressed as average ± standard deviation (SD) (*n* = 3). PE: phycoerythrin; PC: phycocyanin; Scyt: scytonemin. Different letters indicate significant differences among species of cyanobacteria and red algae, analyzed separately (ANOVA, *p* < 0.05, SNK test).

Species	PE	PC	Scyt.
*N. commune*	14.01 ± 0.14 ^e^	24.82 ± 0.07 ^e^	0.31 ± 0.02 ^a^
*Anabaena* sp.	1.86 ± 0.01 ^c^	1.77 ± 0.01 ^b^	0.09 ± 0.04 ^a^
*Scytonema* sp.	3.77 ± 0.01 ^d^	19.58 ± 0.28 ^d^	2.22 ± 0.26 ^c^
*C. scopulorum*	0.78 ± 0.08 ^a^	0.75 ± 0.16 ^a^	0.14 ± 0.02 ^a^
*Lyngbya* sp.	1.21 ± 0.09 ^b^	12.35 ± 0.4 ^c^	1.24 ± 0.18 ^b^
*P. umbilicalis*	3.36 ± 0.04 ^e^	0.25 ± 0.06 ^cd^	-
*G. longissima*	0.51 ± 0.02 ^b^	0.01 ± 0.01 ^a^	-
*G. corneum*	1.25± 0.08 ^d^	0.33 ± 0.10 ^d^	-
*O. pinnatifida*	0.07 ± 0.05 ^a^	0.18 ± 0.02 ^bc^	-
*C. rubrum*	0.91 ± 0.01 ^c^	0.08 ± 0.01 ^ab^	-

**Table 3:** Total content (mg g^−1^ DW) and composition (relative %) of mycosporine-like amino acids (MAAs) in cyanobacteria and red macroalgae species. Values are expressed as average ± standard deviation (SD). Mass spectrometry characterization is also present in the table. Different letters indicate significant differences among the total MAAs content in the different species of cyanobacteria and red algae (ANOVA, *p* < 0.05, SNK test).

Species	Total MAAs Content (mg g^−1^ DW)	Type of MAA	% Relative	Molecular Formula	λ_max_ (nm)	Error (ppm)	Calculated m/z [M + H]^+^	Observed m/z [M + H]^+^
**Cyanobacteria**								
*Scytonema* sp.	0.62 ±0.12 ^a^	Myc-glutaminol	≥98	C_13_H_22_N_2_O_6_	310	3.06	303.15506	303.15414
*Lyngbya* sp.	1.83 ± 0.15 ^b^	Palythine	2.4 ± 0.8	C_10_H_16_N_2_O_5_	320	2.29	245.11320	245.11264
		Asterina-330	96.4 ± 3.2	C_2_H_20_N_2_O_6_	330	2.66	289.13941	289.13864
**Rhodophyta**								
*P. umbilicalis*	5.2 ± 0.40 ^d^	Myc-glutamine	≤1	C_13_H_20_N_2_O_7_	310	1.23	317.13433	317.13394
		Palythine	≤1	C_10_H_16_N_2_O_5_	320	1.14	245.11320	245.11292
		Palythinol	≤1	C_13_H_22_N_2_O_6_	332	1.50	303.15506	303.15460
		Asterina-330	≤1	C_12_H_20_N_2_O_6_	330	1.28	289.13941	289.13904
		Shinorine	8.4 ± 1.2	C_13_H_20_N_2_O_8_	333	1.65	333.12924	333.12869
		Porphyra-334	88.2 ± 2.4	C_14_H_22_N_2_O_8_	334	2.27	347.14489	347.14410
*G. longissima*	0.42 ± 0.10 ^a^	Shinorine	≥98	C_13_H_20_N_2_O_8_	333–334	3.48	333.12924	333.12808
*G. corneum*	1.84 ± 0.23 ^c^	Palythine	14.5 ± 1.1	C_10_H_16_N_2_O_5_	320	2.53	245.11320	245.11258
		Asterina-330	62.9 ± 3.8	C_12_H2_0_N_2_O_6_	330	3.18	289.13941	289.13849
		Shinorine	18.7 ± 1.6	C_13_H_20_N_2_O_8_	333–334	2.85	333.12924	333.12830
*O. pinnatifida*	1.51 ± 0.20 ^bc^	Palythine	28.8 ± 3.3	C_10_H_16_N_2_O_5_	320	1.35	245.11320	245.11287
		Palythinol	4.3 ± 1.2	C_13_H_22_N_2_O_6_	332	1.32	303.15506	303.15466
		Asterina-330	36.1 ± 2.4	C_12_H_20_N_2_O_6_	330	1.62	289.13941	289.13895
		Shinorine	3.8 ± 1.0	C_13_H_20_N_2_O_8_	333–334	1.83	333.12924	333.12863
		Porphyra-334	25.5 ± 1.5	C_14_H_22_N_2_O_8_	334	1.93	347.14489	347.14423
*C. rubrum*	0.97 ± 0.17 ^b^	Palythine	23.3 ± 4.1	C_10_H_16_N_2_O_5_	320	3.38	245.11320	245.11237
		Shinorine	75.8 ± 3.7	C_13_H_20_N_2_O_8_	333–334	3.57	333.12924	333.12805

**Table 4:** Sun protection factor (SPF), UV-A protection factor (UVAPF) and biological effective protection factors (BEPFs) against elastosis and photoaging and % of effective solar absorpted radiation (ESAR) against the different action spectra (erythema, PPD, elastosis and photoaging) of the different creams (base cream, creams including algae extracts and control cream). Data are expressed as average ± standard deviation (SD) (*n* = 4). Different letters indicate significant differences between species and base cream (ANOVA, *p* < 0.05, SNK test).

Action Spectra	Index	Base Cream	*P. umbilicalis*	*Scytonema* sp.	Positive Control (SPF 10)
Erythema (SPF)	BEPFs	1.51 ± 0.02 ^a^	2.12 ± 0.11 ^c^	1.96 ± 0.06 ^bc^	8.30 ± 0.40
	% ESAR	33.40 ± 0.02 ^a^	50.86 ± 2.31 ^c^	47.69 ± 1.37 ^bc^	86.19 ± 4.02
PPD (UVAPF)	BEPFs	1.05 ± 0.01 ^a^	1.45 ± 0.02 ^c^	1.12 ± 0.01 ^b^	7.17 ± 0.20
	% ESAR	3.94 ± 0.16 ^a^	23.69 ± 0.78 ^c^	8.50 ± 0.28 ^b^	81.33 ± 3.67
Elastosis	BEPFs	1.12 ± 0.04 ^a^	1.54 ± 0.03 ^c^	1.26 ± 0.01 ^b^	7.38 ± 0.20
	% ESAR	7.49 ± 0.12 ^a^	24.44 ± 0.96 ^c^	13.26 ± 0.43 ^b^	79.92 ± 2.43
Photoaging	BEPFs	1.03 ± 0.01 ^a^	1.35 ± 0.01 ^c^	1.07 ± 0.01 ^b^	7.15 ± 0.21
	% ESAR	1.05 ± 0.11 ^a^	17.04 ± 0.65 ^c^	4.01 ± 0.12 ^b^	85.27 ± 3.88

**Table 5:** Species of cyanobacteria and red macroalgae used in this study. Samples of cyanobacteria were obtained from the Culture Collection at the Spanish Bank of Algae (BEA), and red algae were collected from the marine coastal environment. BEA codes for the cyanobacteria strains are presented, as well as the dates and places when and where red algae were sampled.

Cyanobacteria		Rhodophyta	
Species	Code	Species	Place and Date of Collection
*Nostoc commune* Vaucher ex Bornet & Flahault	BEA 0024B	*Porphyra umbilicalis* Kützing	“La Caleta” beach ^A^ Tarifa (Cadiz). February 2018
*Anabaena* sp.	BEA 0871B	*Gracilariopsis longissima* (S.G.Gmelin) Steentorft, L.M. Irvine & Farnham	“La Esperanza” saline ^B^ Cadiz. March 2019
*Scytonema* sp.	BEA 1603B	*Gelidium corneum* (Hudson) J.V. Lamouroux	“Las Palomas” island ^C^ Tarifa (Cadiz). October 2019
*Calothrix scopulorum* C. Agardh ex Bornet & Flahault	BEA 0796B	*Osmundea pinnatifida* (Hudson) Stackhouse	“La Araña” beach ^D^ Malaga. February 2018
*Lyngbya* sp.	BEA 1328B	*Ceramium rubrum* C.Agardh	“La Araña” beach ^D^ Malaga. February 2018

A. 36°00′41.7″ N/05°35′59.3″ W; B. 36°30′17.7″ N/06°09′53.1″ W; C. 36°00′01.9″ N/05°36′33.2″ W; D. 36°42′41.3″ N/04°19′38.2″ W.

## Supplementary Materials

The following are available online at https://www.mdpi.com/1660-3397/18/12/659/s1, Table S1: ANOVA summary Tables obtained after the comparison of (A) biochemical composition of the different species (cyanobacteria and Rhodophyta separately), (B) phenolic compounds content extracted in each solvent, (C) antioxidant activity in the different solvents and using the two methods and (D) photoprotective capacity among species. DF: degrees of freedom. Figure S1: HPLC normalized chromatograms of MAAs identified in the different analyzed species: (A) *Scytonema* sp., (B) *Lyngbya* sp., (C) *P. umbilicalis*, (D) *G. longissima*, (E) *G. corneum*, (F) *O. pinnatifida*, (G) *C. rubrum*. Figure S2: Mass spectra of the MAA identified in Scytonema sp.: Mycosporine-glutaminol. Figure S3: Mass spectra of the MAAs identified in *Lyngbya* sp.: (A) Palythine and (B) Asterine-330. Figure S4: Mass spectra of the MAAs identified in *P. umbilicalis*.: (A) Myc-glutamine, (B) Palythine, (C) Palythinol, (D) Asterine-330, (E) Shinorine and (F) Porphyra-334. Figure S5: Mass spectra of the MAAs identified in *G. longissima*.: (A) Shinorine. Figure S6: Mass spectra of the MAAs identified in *G. corneum*.: (A) Palythine, (B) Asterina-330 and (C) Shinorine. Figure S7: Mass spectra of the MAAs identified in *O. pinnatifida*.: (A) Palythine, (B) Palythinol, (C) Asterina-330, (D) Shinorine and (E) Porphyra-334. Figure S8: Mass spectra of the MAAs identified in *C. rubrum*.: (A) Palythine and (B) Shinorine. Table S2: Pearson coefficient (r) between the different variables analyzed in cyanobacteria. Table S3: Pearson coefficient (r) between the different variables analyzed in red macroalgae. Table S4: Pearson coefficient (r) between the PC axis and the analyzed variables in both group of organism tested.
