# Supplementary material for: Cyanobacteria and Red Macroalgae as Potential Sources of Antioxidants and UV Radiation-Absorbing Compounds for Cosmeceutical Applications
Source: Mar Drugs. 2020 Dec 21;18(12):659. doi: 10.3390/md18120659 (PMC7767163; doi:10.3390/md18120659)
Supplement: Supplementary file 1 [file marinedrugs-18-00659-s001.pdf]

Table S.1. ANOVA summary tables obtained after the comparison of: A) Biochemical composition of the different species (Cyanobacteria and Rhodophyta separately). B) Phenolic compounds content extracted in each solvent. C) Antioxidant activity in the different solvents and using the two methods and D) Photoprotective capacity among species. DF: Degrees of freedom.

A)

Content of:	Source of variability	DF	F	p-value
C	Cyanobacteria	4	15846.46	<0.01
	Rhodophyta	4	132.53	<0.01
N	Cyanobacteria	4	36666.20	<0.01
	Rhodophyta	4	45.15	<0.01
C:N	Cyanobacteria	4	24648.35	<0.01
	Rhodophyta	4	44.84	<0.01
Carbohydrates	Cyanobacteria	4	88.10	<0.01
	Rhodophyta	4	32.62	<0.01
Lipids	Cyanobacteria	4	24.09	<0.01
	Rhodophyta	4	61.49	<0.01
Total proteins	Cyanobacteria	4	36666.20	<0.01
	Rhodophyta	4	45.15	<0.01
Soluble proteins	Cyanobacteria	4	1121.17	<0.01
	Rhodophyta	4	413.94	<0.01
Phenolic compounds H_2O_d	Cyanobacteria	4	1313.88	<0.01
	Rhodophyta	4	1250.78	<0.01
Phenolic compounds Ethanol:H_2O_d (1:1)	Cyanobacteria	4	514.03	<0.01
	Rhodophyta	4	2147.31	<0.01
Phenolic compounds Ethanol:H_2O_d (4:1)	Cyanobacteria	4	128.17	<0.01
	Rhodophyta	4	347.43	<0.01
Phenolic compounds Ethanol	Cyanobacteria	4	434.32	<0.01
	Rhodophyta	4	353.95	<0.01
Phycoerithrin	Cyanobacteria	4	13243.98	<0.01
	Rhodophyta	4	412.520	<0.01
Phycocianin	Cyanobacteria	4	6276.09	<0.01
	Rhodophyta	4	15.39	<0.01
Chlorophyll	Cyanobacteria	4	74.48	<0.01
	Rhodophyta	4	79.95	<0.01
Carotenoids	Cyanobacteria	4	123.46	<0.01
	Rhodophyta	4	74.48	<0.01
Scytonemin	Cyanobacteria	4	119.35	<0.01
MAAs	Cyanobacteria	4	63.77	<0.01
	Rhodophyta	4	139.76	<0.01
ABTS H_2O_d	Cyanobacteria	4	549.11	<0.01
	Rhodophyta	4	159.10	<0.01
ABTS Ethanol:H_2O_d (1:1)	Cyanobacteria	4	679.95	<0.01
	Rhodophyta	4	1031.99	<0.01
ABTS Ethanol:H_2O_d (4:1)	Cyanobacteria	4	250.46	<0.01
	Rhodophyta	4	153.35	<0.01
ABTS Ethanol	Cyanobacteria	4	202.92	<0.01
	Rhodophyta	4	120.63	<0.01
DPPH	Cyanobacteria	4	471.29	<0.01

	H_2O_d	Rhodophyta	4	1115.21	<0.01
	DPPH Ethanol:H_2O_d (1:1)	Cyanobacteria	4	17.14	<0.01
		Rhodophyta	4	827.81	<0.01
	DPPH Ethanol:H_2O_d (4:1)	Cyanobacteria	4	17.13	<0.01
		Rhodophyta	4	467.33	<0.01
	DPPH Ethanol	Cyanobacteria	4	2171.57	<0.01
		Rhodophyta	4	94.15	<0.01
**B)**	**Phenolic compounds content of:**				
	P. umbilicalis	Solvent	3	506.22	<0.01
	G. longissima	Solvent	3	769.67	<0.01
	G. corneun	Solvent	3	421.69	<0.01
	O. pinnatifida	Solvent	3	452.50	<0.01
	C. rubrum	Solvent	3	726.49	<0.01
	N. commune	Solvent	3	726.49	<0.01
	Anabaena sp.	Solvent	3	605.45	<0.01
	Scytonema sp.	Solvent	3	27939.01	<0.01
	C. scopulorum	Solvent	3	1548.54	<0.01
	Lyngbya sp.	Solvent	3	99.63	<0.01
**C)**	**Antioxidant activity of:**	**Source of variability**	**GL**	**F**	**p-value**
	P. umbilicalis	Solvent	3	655.99	<0.01
		Method	1	2974.07	<0.01
		Solvent*Method	3	213.47	<0.01
	G. longissima	Solvent	3	451.37	<0.01
		Method	1	10174.34	<0.01
		Solvent*Method	3	320.38	<0.01
	C. corneus	Solvent	3	131.12	<0.01
		Method	1	929.07	<0.01
		Solvent*Method	3	34.35	<0.01
	G. corneun	Solvent	3	151.47	<0.01
		Method	1	437.39	<0.01
		Solvent*Method	3	69.85	<0.01
	O. pinnatifida	Solvent	3	127.80	<0.01
		Method	1	2820.90	<0.01
		Solvent*Method	3	57.96	<0.01
	C. rubrum	Solvent	3	598.14	<0.01
		Method	1	4479.84	<0.01
		Solvent*Method	3	131.84	<0.01
	N. commune	Solvent	3	843.94	<0.01
		Method	1	800.41	<0.01
		Solvent*Method	3	89.44	<0.01
	Anabaena sp. (ABTS)	Solvent	3	216.32	<0.01
	Scytonema sp.	Solvent	3	276.61	<0.01
		Method	1	1497.53	<0.01
		Solvent*Method	3	49.29	<0.01
	C. scopulorum	Solvent	3	1449.44	<0.01
		Method	1	7828.93	<0.01
		Solvent*Method	3	60.81	<0.01
	Lyngbya sp.	Solvent	3	264.39	<0.01

		Method	1	458.32	<0.01
		Solvent*Method	3	28.17	<0.01
**D)**	**BEPFS**				
	Erythema	Species	2	31.18	<0.01
	PPD	Species	2	433.22	<0.01
	Elastosis	Species	2	131.66	<0.01
	Photoaging	Species	2	620.28	<0.01
	**ESAR**				
	Erythema	Species	2	34.70	<0.01
	PPD	Species	2	315.78	<0.01
	Elastosis	Species	2	145.57	<0.01
	Photoaging	Species	2	620.28	<0.01

Figure S.1. HPLC normalized chromatograms of MAAs identified in the different analysed species: A) *Scytonema* sp., B) *Lyngbya* sp., C) *P. umbilicalis*, D) *G. longissima*, E) *G. corneum*, F) *O. pinnatifida*, G) *C. rubrum*.

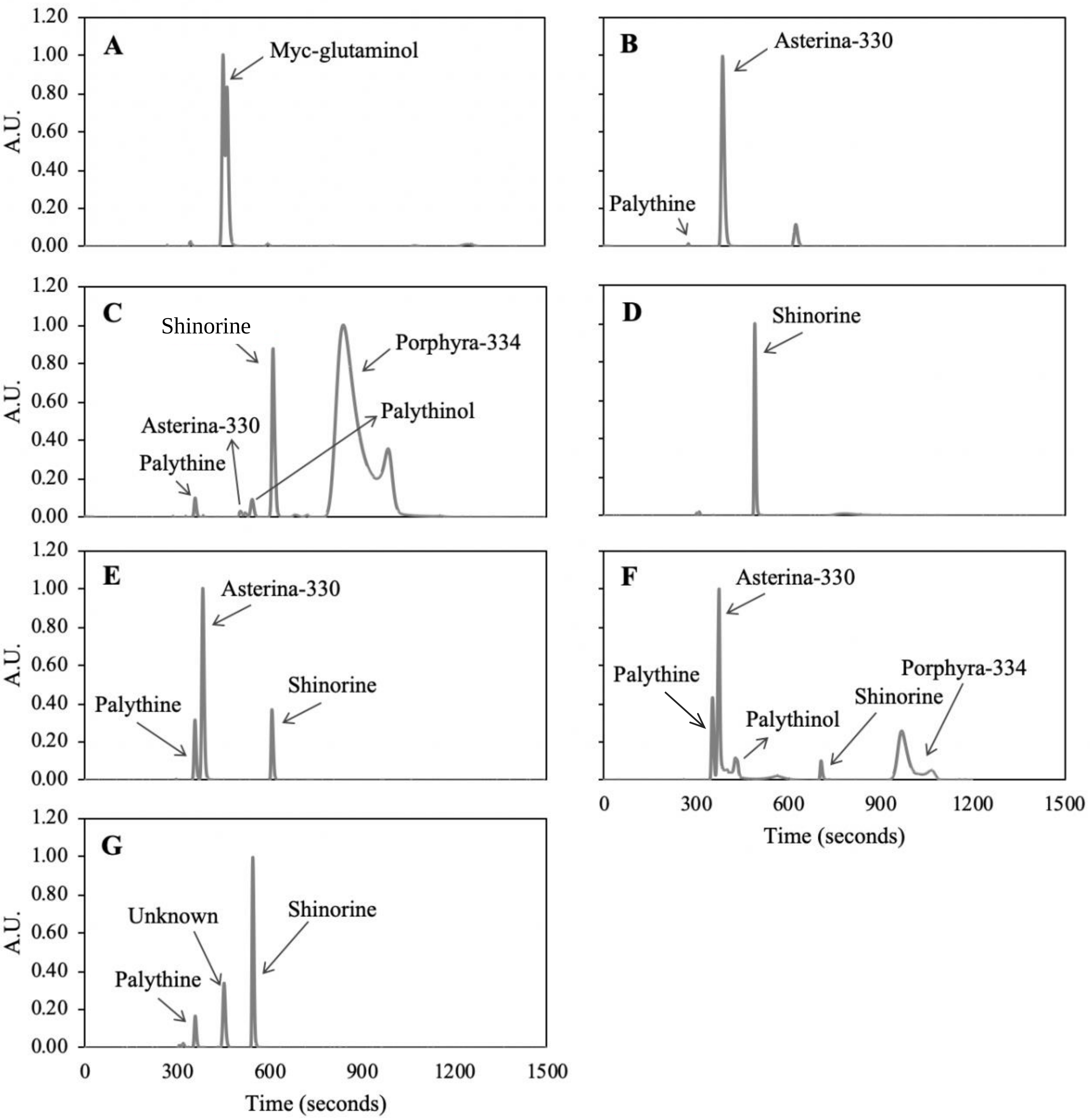

Figure S.2. Mass spectra of the MAA identified in *Scytonema* sp.: Mycosporine-glutaminol

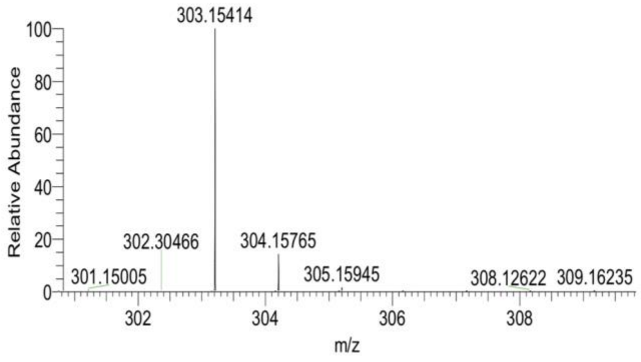

Figure S.3. Mass spectra of the MAAs identified in *Lyngbya* sp.: A) Palythine and B) Asterine-330.

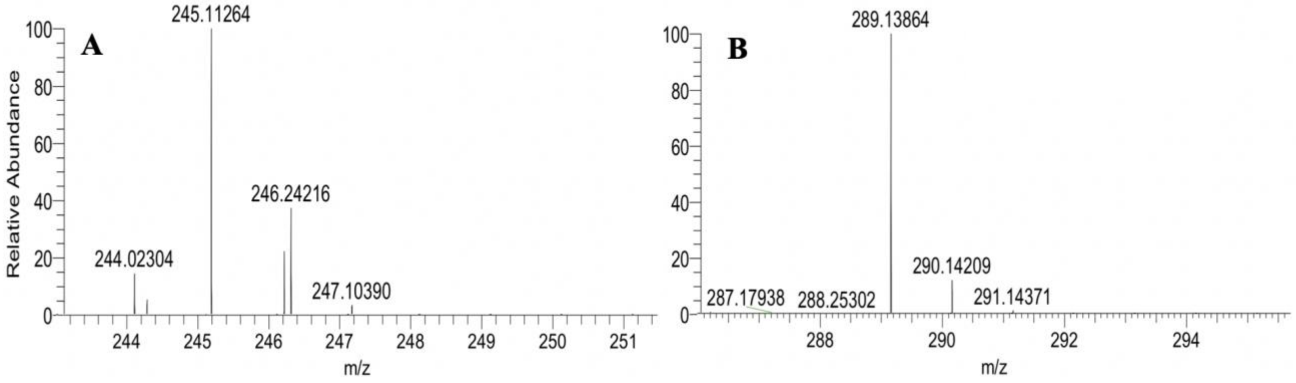

Figure S.4. Mass spectra of the MAAs identified in *P. umbilicalis*.: A) Myc-glutamine, B) Palythine, C) Palythinol, D) Asterine-330, E) Shinorine and F) Porphyra-334

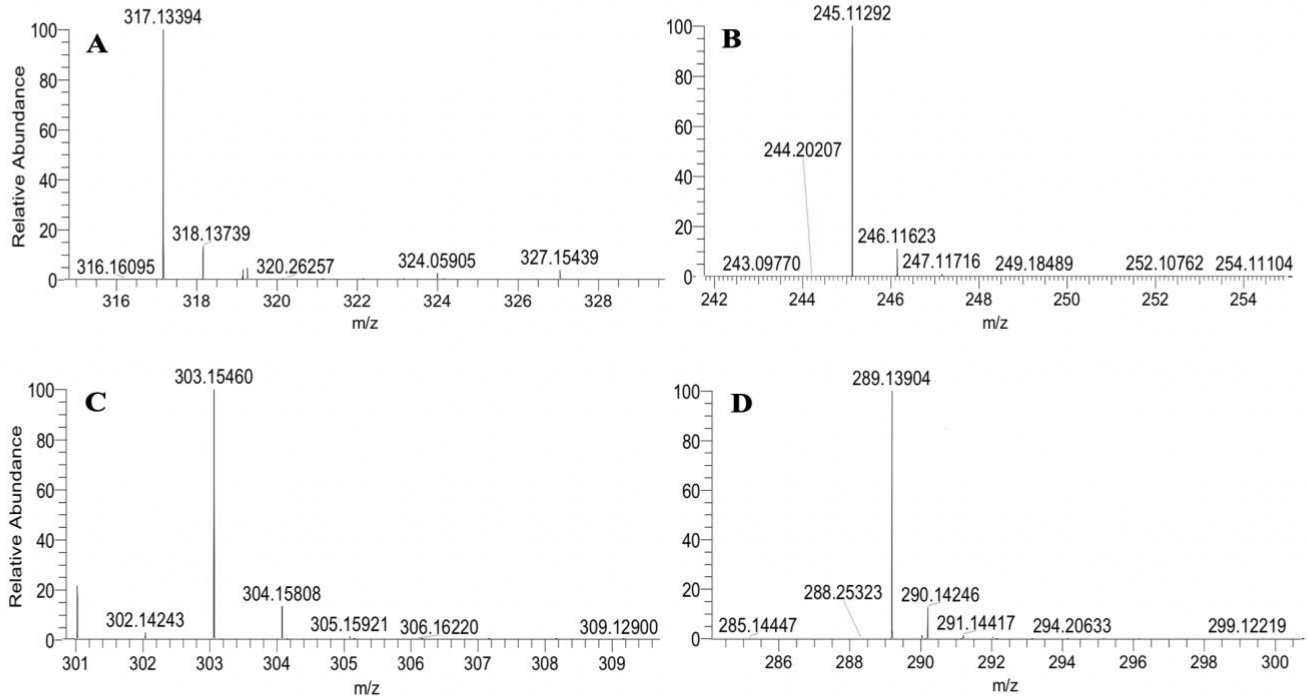

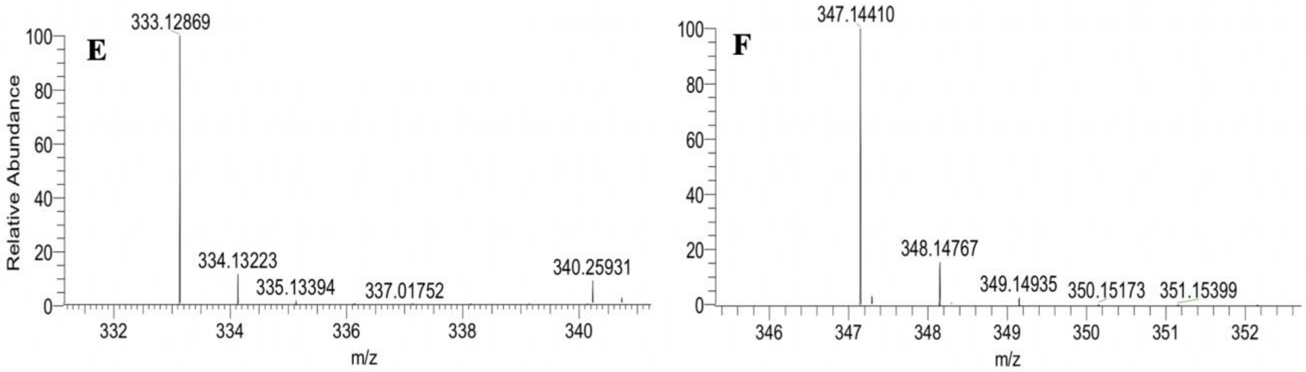

Figure S.5. Mass spectra of the MAAs identified in *G. longissima*.: Shinorine

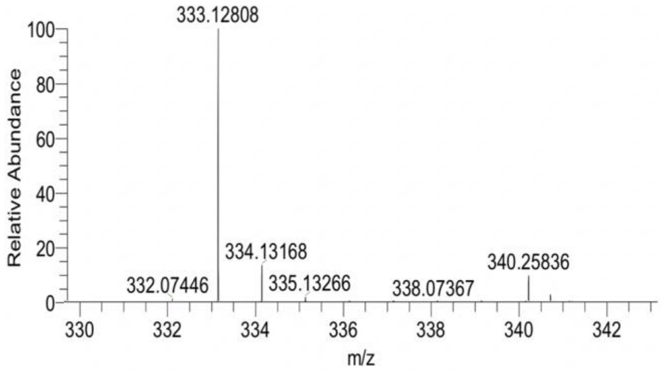

Figure S.6. Mass spectra of the MAAs identified in *G. corneum*.: A) Palythine, B) Asterina-330 and C) Shinorine.

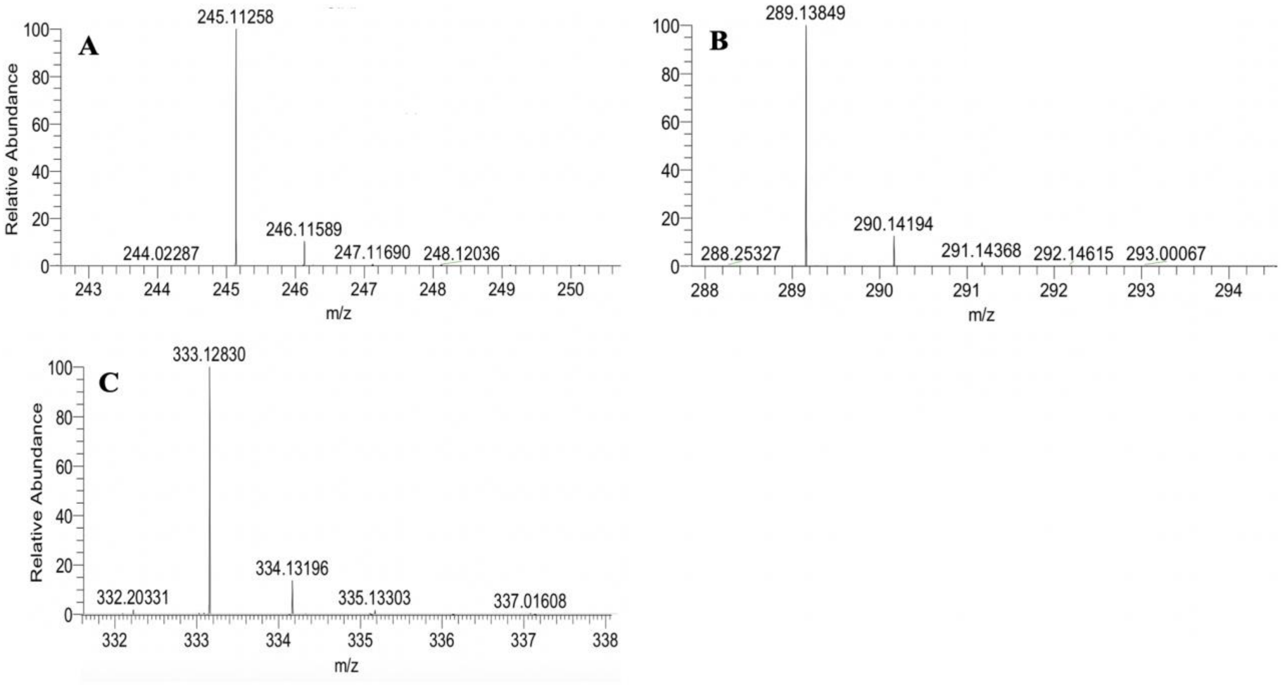

Figure S.7. Mass spectra of the MAAs identified in *O. pinnatifida*.: A) Palythine, B) Palythinol, C) Asterina-330, D) Shinorine and E) Porphyra-334.

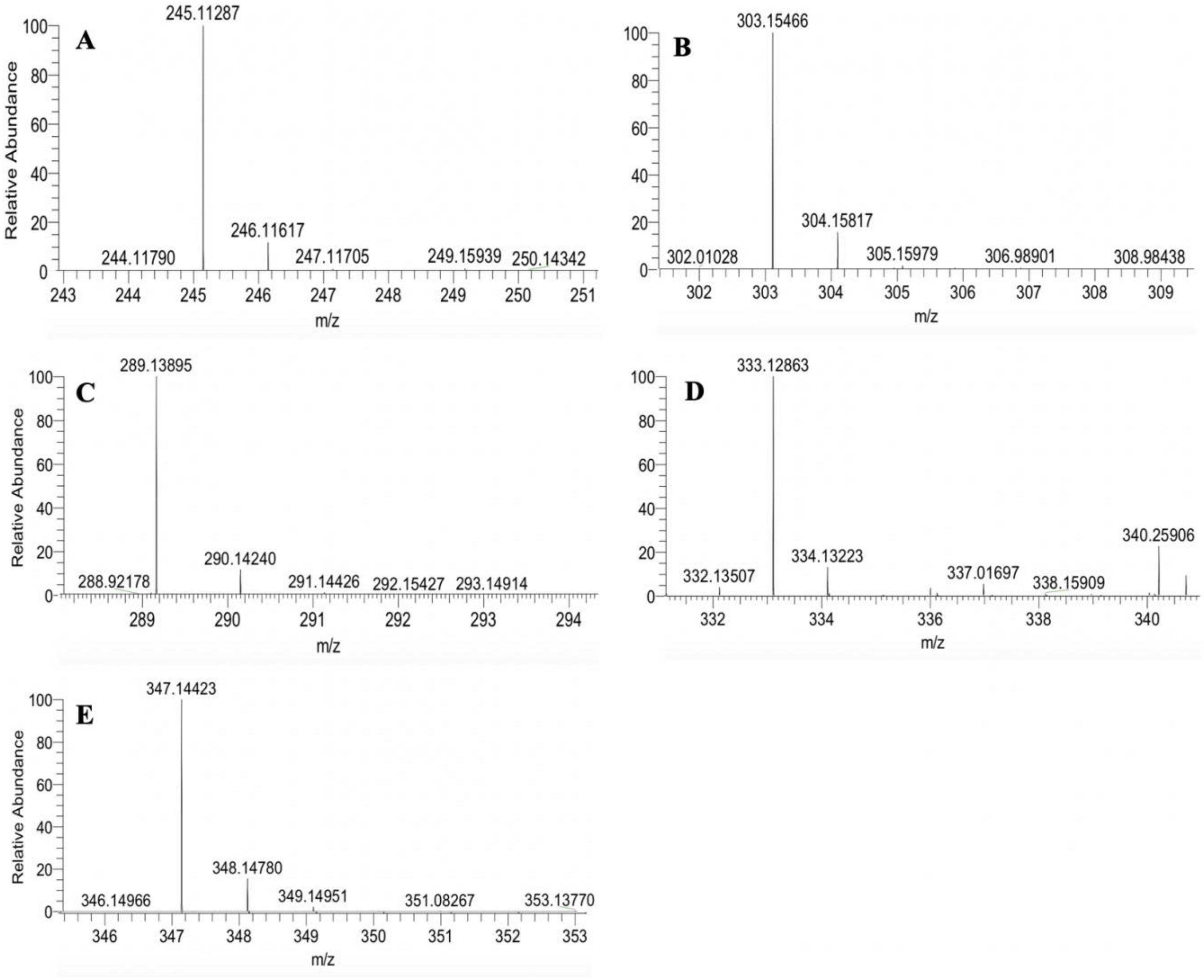

Figure S.8. Mass spectra of the MAAs identified in *C. rubrum*.: A) Palythine and B) Shinorine

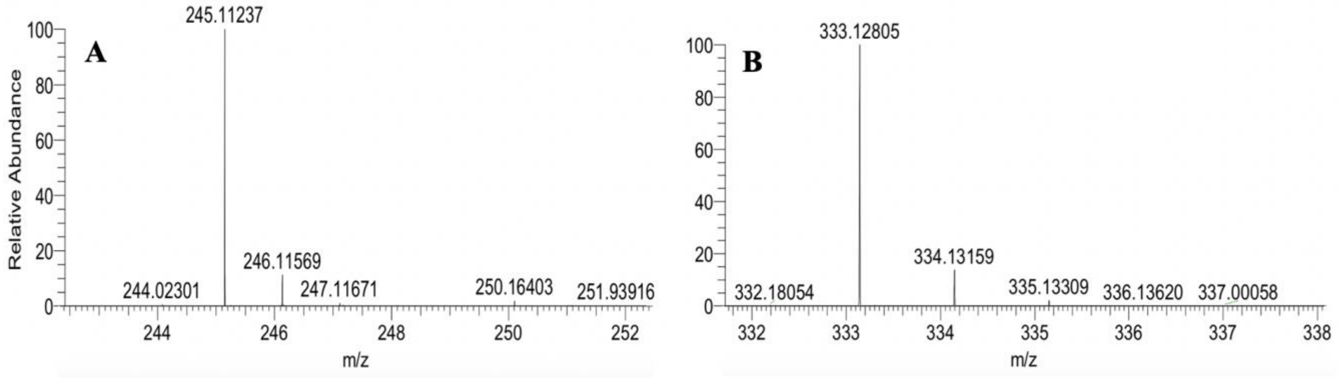

Table S.2. Pearson coefficient (r) between the different variables analyzed in cyanobacteria

	ABTS.1	ABTS.2	ABTS.3	ABTS.4	DPPH.1	DPPH.2	DPPH.3	DPPH.4	C	N	C:N	TP	SP	Carboh.	Lipids	Phenols.1	Phenols.2	Phenols.3	Phenols.4	PE	PC	Scy.	MAAs
**ABTS.1**	-																						
**ABTS.2**	0.980**	-																					
**ABTS.3**	0.754**	0.849**	-																				
**ABTS.4**	0.885**	0.941**	0.898**	-																			
**DPPH.1**	0.875**	0.782**	0.372	0.582*	-																		
**DPPH.2**	0.699**	0.602*	0.241	0.403	0.886**	-																	
**DPPH.3**	0.405	0.410	0.182	0.334	0.498	0.655**	-																
**DPPH.4**	0.648**	0.682**	0.458	0.674**	0.584*	0.607*	0.877**	-															
**C**	0.758**	0.758**	0.786**	0.633*	0.598*	0.568*	0.135	0.238	-														
**N**	0.358	0.432	0.768**	0.468	0.006	-0.048	-0.328	-0.172	0.772**	-													
**C:N**	-0.309	-0.392	-0.755**	-0.441	0.052	0.089	0.332	0.184	-0.741**	-0.998**	-												
**TP**	0.358	0.432	0.768**	0.468	0.006	-0.048	-0.328	-0.172	0.772**	1.000**	-0.997**	-											
**SP**	0.662**	0.630*	0.539*	0.428	0.672**	0.785**	0.440	0.398	0.881**	0.453	-0.426	0.453	-										
**Carboh.**	0.635*	0.497	0.087	0.289	0.820**	0.686**	0.101	0.199	0.428	-0.045	0.104	-0.045	0.471	-									
**Lipids**	0.024	-0.143	-0.543*	-0.376	0.464	0.538*	0.171	-0.021	-0.056	-0.533*	0.566*	-0.533*	0.258	0.611*	-								
**Phenols.1**	0.983**	0.972**	0.754*	0.867**	0.875**	0.763*	0.519*	0.726**	0.775**	0.333	-0.289	0.333	0.744**	0.589*	0.053	-							
**Phenols.2**	0.711**	0.773**	0.949**	0.789**	0.367	0.269	0.002	0.235	0.896**	0.897**	-0.879**	0.897**	0.625*	0.176	-0.442	0.699**	-						
**Phenols.3**	0.765**	0.850**	0.928**	0.876**	0.448	0.439	0.435	0.673**	0.754**	0.598*	-0.588*	0.598*	0.659**	0.104	-0.398	0.817**	0.845**	-					
**Phenols.4**	0.742**	0.808**	0.713*	0.850**	0.538*	0.524*	0.738**	0.943**	0.431	0.138	-0.127	0.138	0.458	0.133	-0.246	0.804**	0.516*	0.856**	-				
**PE**	0.283	0.153	-0.049	-0.135	0.549*	0.620*	0.013	-0.171	0.569*	0.171	-0.133	0.171	0.714**	0.634*	0.685**	0.307	0.172	-0.013	-0.213	-			
**PC**	0.621*	0.531*	0.225	0.270	0.825**	0.925**	0.581*	0.477	0.639*	0.029	0.008	0.029	0.892**	0.648**	0.601*	0.703**	0.278	0.405	0.397	0.781**	-		
**Scy.**	0.730**	0.771**	0.551*	0.752**	0.637*	0.585*	0.798**	0.977**	0.322	-0.086	0.105	-0.086	0.359	0.241	-0.041	0.796**	0.330	0.723**	0.951**	-0.135	0.490	-	
**MAAs**	-0.094	-0.024	-0.011	0.028	-0.087	0.193	0.789**	0.645**	-0.208	-0.356	0.321	-0.356	0.102	-0.427	-0.091	0.049	-0.209	0.265	0.524*	-0.337	0.152	0.535	-

ABTS.1: ABTS assay in H_2O_d extraction; **ABTS.2:** ABTS assay in ethanol: H_2O_d (1:1) extraction; **ABTS.3:** ABTS assay in ethanol:H_2O_d (4:1); **ABTS.4:** ABTS assay in ethanol extraction; **DPPH.1:** DPPH assay in H_2O_d extraction; **DPPH.2:** DPPH assay in ethanol: H_2O_d (1:1); **DPPH.3:** DPPH assay in ethanol:H_2O_d (4:1); **DPPH.4:** DPPH assay in ethanol extraction; **C:** Total carbon content; **N:** Total nitrogen content; **C:N:** Carbon:Nitrogen ratio; **TP:** Total proteins; **SP:** Soluble proteins; **Carboh.:** Carbohydrates; **Phenols.1:** Phenols content in H_2O_d extraction; **Phenols.2:** Phenols content in ethanol: H_2O_d (1:1) extraction; **Phenols.3:** Phenols content in ethanol: H_2O_d (4:1); **Phenols.4:** Phenols content in ethanol extraction; **PE:** Phycoerythrin; **PC:** Phycocianin; **Scy.:** Scytonemin; **MAAs:** Mycosporine like aminoacids

Table S.3. Pearson coefficient (r) between the different variables analyzed in red macroalgae

	ABTS.1	ABTS.2	ABTS.3	ABTS.4	DPPH.1	DPPH.2	DPPH.3	DPPH.4	C	N	C:N	TP	SP	Carboh.	Lipids	Phenols.1	Phenols.2	Phenols.3	Phenols.4	PE	PC	MAAs
**ABTS.1**	-																					
**ABTS.2**	0.936**	-																				
**ABTS.3**	.585*	0.805**	-																			
**ABTS.4**	0.179	0.497*	0.842**	-																		
**DPPH.1**	0.138	0.022	-0.215	-0.183	-																	
**DPPH.2**	0.325	0.219	-0.058	-0.114	0.976**	-																
**DPPH.3**	0.152	0.109	0.127	0.033	0.804**	0.819**	-															
**DPPH.4**	-0.022	0.112	0.458	0.482	0.383	0.389	0.796**	-														
**C**	0.213	-0.020	-0.244	-0.649**	-0.333	-0.289	-0.202	-0.368	-													
**N**	0.514*	0.219	-0.352	-0.689**	0.315	0.371	0.050	-0.465	0.623*	-												
**C:N**	-0.329	-0.233	0.195	0.143	-0.752**	-0.763**	-0.290	0.153	0.359	-0.503	-											
**TP**	0.514*	0.219	-0.352	-0.689**	0.316	0.371	0.050	-0.465	0.623*	1.000**	-0.503	-										
**SP**	0.696**	0.427	-0.168	-0.534*	0.354	0.450	0.084	-0.436	0.488	0.936**	-0.562*	0.936**	-									
**Carboh.**	0.439	0.284	0.107	-0.407	-0.488	-0.381	-0.441	-0.558*	-0.833**	0.578*	0.232	0.577*	0.543*	-								
**Lipids**	0.528*	0.778**	0.903**	0.877**	0.006	0.145	0.192	0.445	-0.476	-0.353	-0.067	-0.353	-0.147	-0.184	-							
**Phenols.1**	0.951**	0.976**	0.675	0.392	0.126	0.145	0.086	-0.009	-0.033	0.346	-0.399	0.346	0.557*	0.282	0.711**	-						
**Phenols.2**	0.806**	0.958**	0.908**	0.711**	-0.001	0.177	0.141	0.277	-0.235	-0.045	-0.156	-0.045	0.175	0.081	0.918**	0.908**	-					
**Phenols.3**	0.679**	0.889**	0.964**	0.812**	-0.127	0.043	0.097	0.349	-0.283	-0.234	0.013	-0.234	-0.023	0.036	0.955**	0.804**	0.976**	-				
**Phenols.4**	0.420	0.694**	0.961**	0.922**	-0.327	-0.202	-0.020	0.413	-0.345	-0.496	0.251	-0.496	-0.343	-0.075	0.908**	0.567*	0.845**	0.930**	-			
**PE**	0.576**	0.302	-0.194	-0.639*	-0.013	0.082	-0.125	-0.521*	0.827**	0.908**	-0.159	0.907**	0.885**	0.820**	-0.300	0.376	0.031	-0.113	-0.350	-		
**PC**	0.379	0.297	0.255	-0.173	0.253	-0.153	0.127	0.142	0.771**	0.356	0.429	0.356	0.259	0.634*	0.059	0.206	0.195	0.191	0.161	0.564*	-	
**MAAs**	0.809**	0.634*	0.182	-0.272	-0.090	0.067	-0.156	-0.445	0.629*	0.754*	-0.169	0.754**	0.838**	0.763*	0.058	0.675**	0.399	0.269	0.012	0.893**	0.478	-

ABTS.1: ABTS assay in $_{d}H_2O$ extraction; **ABTS.2:** ABTS assay in ethanol:$_{d}H_2O$ (1:1) extraction; **ABTS.3:** ABTS assay in ethanol:$_{d}H_2O$ (4:1); **ABTS.4:** ABTS assay in ethanol extraction; **DPPH.1:** DPPH assay in $_{d}H_2O$ extraction; **DPPH.2:** DPPH assay in ethanol:$_{d}H_2O$ (1:1); **DPPH.3:** DPPH assay in ethanol:$_{d}H_2O$ (4:1); **DPPH.4:** DPPH assay in ethanol extraction; **C:** Total carbon content; **N:** Total nitrogen content; **C:N:** Carbon:Nitrogen ratio; **TP:** Total proteins; **SP:** Soluble proteins; **Carboh.:** Carbohydrates; **Phenols.1:** Phenols content in $_{d}H_2O$ extraction; **Phenols.2:** Phenols content in ethanol:$_{d}H_2O$ (1:1) extraction; **Phenols.3:** Phenols content in ethanol:$_{d}H_2O$ (4:1); **Phenols.4:** Phenols content in ethanol extraction; **PE:** Phycoerythrin; **PC:** Phycocianin; **MAAs:** Mycosporine like aminoacids

Table S.4. Pearson coefficient (r) between the PC axis and the analyzed variables in both group of organism tested.

	ABTS.1	ABTS.2	ABTS.3	ABTS.4	DPPH.1	DPPH.2	DPPH.3	DPPH.4	C	N	S. proteins	Carboh.	Lipids	Phenols.1	Phenols.2	Phenols.3	Phenols.4	PE	PC	Scytonemin	MAAS
**PC1**	0.726	0.583	0.442	0.437	0.680	-0.042	0.102	0.202	0.804	0.482	0.711	0.959	0.524	0.577	0.020	-0.059	0.056	0.709	0.694	0.438	0.016
**PC2**	0.277	0.207	0.640	0.418	0.039	-0.257	0.189	0.180	0.590	0.742	0.483	-0.284	0.444	0.067	-0.077	-0.124	0.004	0.081	0.277	0.329	-0.209
**PC3**	-0.500	-0.566	-0.375	-0.463	-0.590	-0.619	-0.567	-0.554	0.071	-0.095	-0.450	0.004	-0.196	-0.660	-0.441	-0.472	-0.527	-0.221	-0.496	-0.486	0.103
**PC4**	-0.269	-0.427	-0.447	-0.478	0.079	0.180	0.400	0.044	0.014	-0.318	0.179	-0.006	0.156	-0.297	-0.534	-0.449	-0.356	0.399	0.362	-0.006	0.091
**PC5**	-0.247	-0.266	-0.179	-0.398	-0.253	0.140	-0.516	-0.709	-0.023	0.315	0.151	0.004	-0.103	-0.159	0.119	-0.107	-0.534	0.361	-0.108	-0.588	0.085

ABTS.1: ABTS assay in $_dH_2O$ extraction; **ABTS.2:** ABTS assay in ethanol:$_dH_2O$ (1:1) extraction; **ABTS.3:** ABTS assay in ethanol:$_dH_2O$ (4:1); **ABTS.4:** ABTS assay in ethanol extraction; **DPPH.1:** DPPH assay in $_dH_2O$ extraction; **DPPH.2:** DPPH assay in ethanol:$_dH_2O$ (1:1); **DPPH.3:** DPPH assay in ethanol:$_dH_2O$ (4:1); **DPPH.4:** DPPH assay in ethanol extraction; **C:** Total carbon content; **N:** Total nitrogen content; **S. proteins:** Soluble proteins; **Carboh.:** Carbohydrates; **Phenols.1:** Phenols content in $_dH_2O$ extraction; **Phenols.2:** Phenols content in ethanol:$_dH_2O$ (1:1) extraction; **Phenols.3:** Phenols content in ethanol:$_dH_2O$ (4:1); **Phenols.4:** Phenols content in ethanol extraction; **PE:** Phycoerythrin; **PC:** Phycocianin; **MAAs:** Mycosporine like aminoacids
